# Real‐Time Behaviour Recognition on Bio‐Loggers Enables Autonomous Audio Playback Experiments in Free‐Ranging Seabirds

**DOI:** 10.1002/ece3.71832

**Published:** 2025-08-06

**Authors:** Ryoma Otsuka, Hibiki Sugiyama, Yuichi Mizutani, Ken Yoda, Takuya Maekawa

**Affiliations:** ^1^ Graduate School of Information Science and Technology The University of Osaka Suita Osaka Japan; ^2^ Graduate School of Environmental Studies Nagoya University Nagoya Aichi Japan; ^3^ Institute for Advanced Co‐Creation Studies The University of Osaka Suita Osaka Japan

**Keywords:** acceleration data, bio‐logging, causal inference, edge computing, machine learning, playback

## Abstract

Audio playback experiments in the natural environment have been a powerful tool in animal behaviour and ecology, revealing causal relationships between animal movements/behaviours and audio stimuli. However, traditional audio playback experiments could only be performed in limited locations and/or situations where direct observation and/or video recording by human observers or installation of automated devices, such as camera traps, were possible. To overcome the limitation, we designed an autonomous audio playback system on bio‐loggers in the natural environment. In this system, an on‐board machine learning model estimates animals' behavioural state (e.g., flying or not) in real time using data from a low‐power accelerometer. If the target behaviour (e.g., flying) is detected and other predefined criteria are met, the logger activates high‐cost sensors, including a video camera, and plays audio from a built‐in speaker. The logger can record fine‐scale behavioural data before, during, and after the playback using multiple modalities (e.g., acceleration, GPS, and video). To examine the validity of the system, we performed field experiments targeting freely ranging black‐tailed gulls ( *Larus crassirostris* ) in Japan. The real‐time behaviour recognition using acceleration data demonstrated high accuracy in the field experiments (macro F1‐score = 0.91). The playback experiments were performed almost perfectly as we intended when birds were flying outside the colony (46 playback events were collected from eight birds), except for several failures due to hardware malfunctions. Using three response indicators (based on acceleration, GPS, and video data), Bayesian statistical modelling and causal inference analysis showed that several birds clearly responded to the audio stimuli, but to both predator call and noise sound. Despite some remaining practical challenges, the results demonstrated a successful proof of concept for the proposed audio playback system on bio‐loggers. By removing the location constraints of traditional playback experiments, the system allows a variety of playback experiments to be tested in various situations. In the future, the system can be extended to stimulate other sensor modalities (e.g., magnetic sensors), expanding the possibilities for intervention methods in the wild environment.

## Graphical abstract

We developed an autonomous audio playback system integrated into bio‐loggers to conduct behavioural experiments in wild animals without location constraints. Field tests with free‐ranging black‐tailed gulls demonstrated accurate real‐time behaviour recognition and successful audio playback events, revealing behavioural responses to audio stimuli. This system could expand the potential for intervention‐based studies in natural environments.

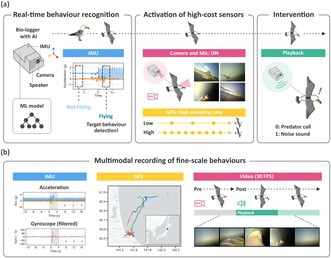

## 1 Introduction

Audio playback experiments have played a pivotal role in understanding animal behaviour and ecology (e.g., Seyfarth et al. 1980; Flower et al. 2014; Suraci et al. 2016). However, a key technical limitation of traditional playback experiments lies in their spatial constraints. Generally, playback experiments in the field are restricted to locations where observers can make direct observations or where video recording is possible. For example, in the case of birds, many experiments have been conducted in the vicinity of roosting sites (e.g., Dibnah et al. 2022) or breeding colonies (e.g., MacLean and Bonter 2013). To enable remote playback systems without human observers, an automated behavioural response (ABR) system that combines camera traps and speakers has been proposed (e.g., Suraci et al. 2017). However, the ABR system still has the following constraints: (1) the locations where cameras can be installed are restricted; (2) cameras are fixed with narrow coverages; and (3) the timing of the playback cannot be flexibly controlled by the device, as a typical ABR system simply plays sound from a speaker connected to a passive infrared motion sensor (e.g., Suraci et al. 2017). These constraints have made it challenging to conduct playback experiments on animals engaging in natural activities in environments beyond human observation, such as foraging in the deep sea or flying through the open sky.

**FIGURE 1:**
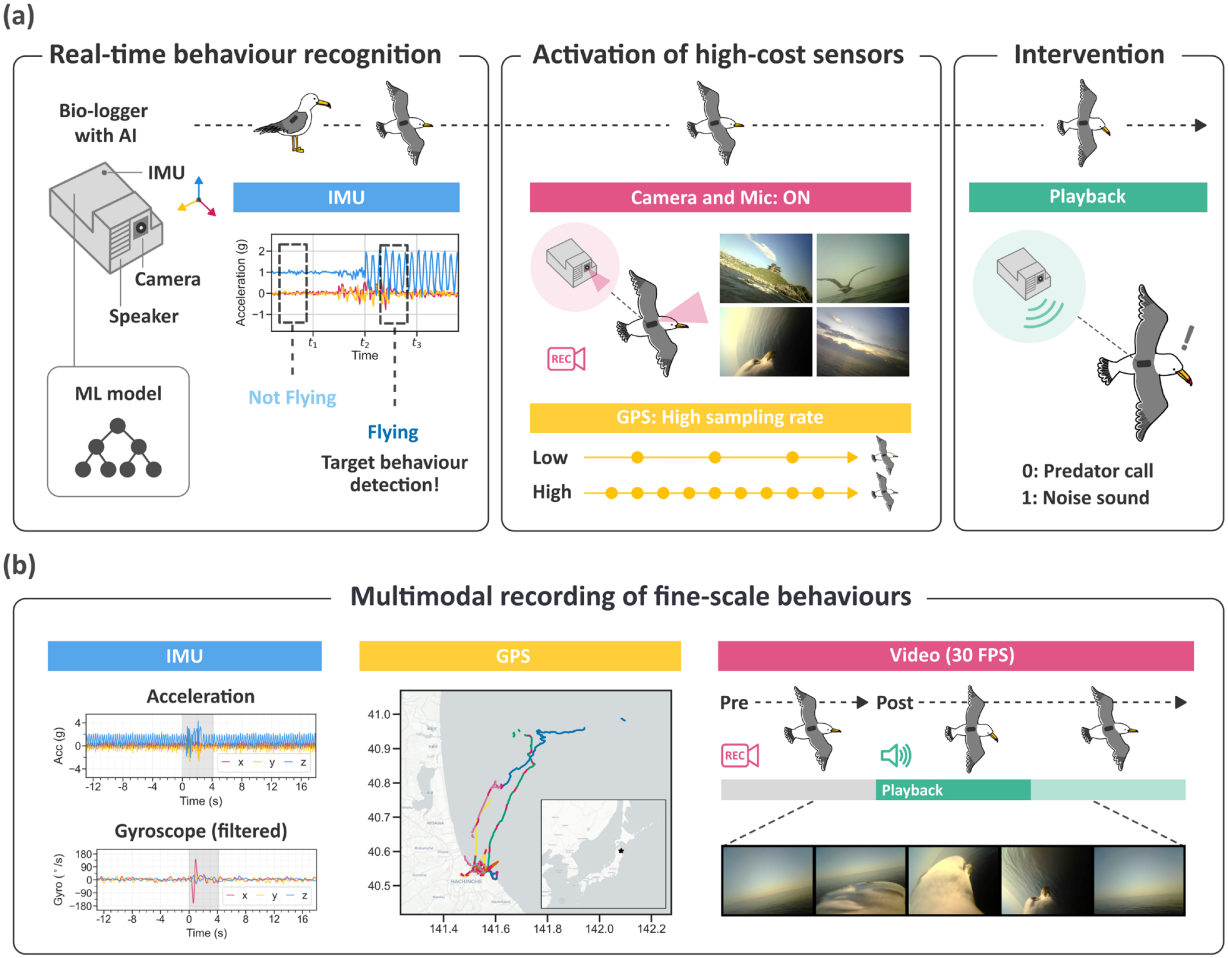
Conceptual overview of autonomous audio playback experiment system using bio‐loggers attached to seabirds (a). The bio‐logger has a real‐time behaviour recognition function using machine learning. When the system detects the target behaviour (e.g., flying), the high‐cost sensors are activated (the camera is turned on and the GPS sampling rate is increased (e.g., 1 Hz)). Then, the bio‐logger performs a playback experiment. The system can record fine‐scale behaviour of before (pre), during and after (post) the playback using multimodal sensors such as accelerometer, GPS, and camera (b). The map in the centre of (b) displays GPS tracking data from eight birds, with each colour representing the tracks of a different individual and red colour representing video recording sessions. The star on the map of Japan marks the location of Kabushima Island.

To overcome the limitations, we propose a playback experiment system on bio‐loggers based on edge computing technology using machine learning. Edge computing is a distributed computing paradigm that brings computation and data storage closer to the data sources, reducing the latency and/or data sizes for wireless transmission (Yu et al. 2024). Utilising this edge computing approach, bio‐loggers can now process data more efficiently in real time, a concept we term AI on Animals (AIoA) (Korpela et al. 2020; Tanigaki et al. 2024). For example, Korpela et al. (2020) achieved efficient video data collection using supervised machine learning models, while Tanigaki et al. (2024) captured rare behaviours using unsupervised outlier detection models. Another research (Yu et al. 2022) used edge computing techniques to compress data that are transmitted through the 3G mobile network. Building upon the concept of these systems, we have developed a bio‐logger that autonomously controls video recording and playback experiments (Figure 1). The bio‐logger can estimate the behavioural state of animals in real time using a machine learning model on‐board. For instance, the bio‐logger can play an audio file (such as a predator call, conspecific vocalisations, environmental sounds, or white noise) from a built‐in speaker when the animal is moving in areas beyond human observation. Furthermore, the bio‐logger can record the animal's fine‐scale behaviours before, during, and after the playback using a variety of data modalities, including acceleration, GPS, and video data to analyse animals' behavioural responses to the audio playbacks. To verify the usefulness of this system, we conducted field experiments, targeting black‐tailed gulls ( *Larus crassirostris* ) in Kabushima Island, Japan.

## 2 Materials and Methods

### 2.1 Logger Design

**FIGURE 2:**
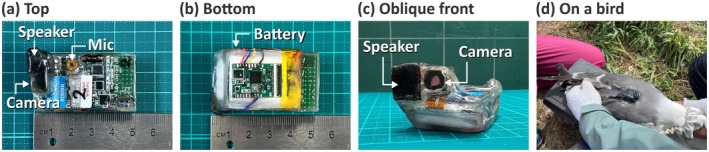
Photos of our AI playback bio‐logger (waterproofed). (a) Top view shows the main board with multiple sensors (GPS, IMU, magnetometer, water depth, illuminance, camera, and microphone) and SD cards. (b) Bottom view shows the 500 mAh battery and extension board for controlling the speaker. (c) Oblique front view shows the camera and speaker and how they were waterproofed. (d) The bio‐logger attached to a bird (head on the right).

The bio‐logger's primary system on a chip (SoC) (Figure 2a) is the nRF52840 (Nordic Semiconductor, Trondheim, Norway), a 32‐bit microcontroller with a 64 MHz clock speed, 1 MB of program memory, and 256 KB of Random Access Memory (RAM). The bio‐logger contains a variety of sensors, including an inertial measurement unit (IMU; BMI270, Bosch Sensortec, Reutlingen, Germany), a magnetometer (BMM150, Bosch Sensortec), and a global positioning system (GPS) module (ZOE‐M8Q‐0, u‐blox, Zurich, Switzerland). Additionally, as illustrated in Figure 2a,c, the bio‐logger is equipped with a video camera (OV2640, OmniVision Technologies, California, USA) capable of recording 30 frames per second at a resolution of 640 × 480, a microphone (ICS‐43434, TDK InvenSense, California, USA), and a speaker (UGCM0903EPD, Universal (Changzhou) Electronics, Jiangsu, China). The camera and microphone are controlled by another SoC, ESP32 (Espressif Systems, Shanghai, China), on the main board. The speaker is controlled by a different SoC, ESP32, on an extension board (Figure 2b), which is connected to the main board and the speaker. The bio‐logger can be combined with batteries of various sizes. In the field experiments described below, a 3.7 V, 500 mAh lithium‐ion polymer battery (Figure 2b) was used. In this case, the bio‐logger's dimensions were 50 mm in length, 28 mm in width, and 15 mm (24 mm up to the camera or speaker top) in height, with a weight ranging from 25.80 to 26.79 g after waterproofing (Text S1).

### 2.2 Autonomous Playback Experiment System on Bio‐Loggers

The system is designed to simultaneously enable audio playback at targeted timings, acquisition of multimodal data (accelerometer, GPS, and video) before and after the intervention, and efficient battery usage. First, we trained a machine learning model (Decision Tree) to classify behaviours (flying or non‐flying) of black‐tailed gulls, using previously collected and annotated acceleration data in a supervised manner. Then, we implemented the model on the bio‐logger (Text S2 and Figure S1). The on‐board machine learning model estimates the bird's behavioural state every second based on low‐power acceleration sensor data. When a target behaviour (i.e., flying in this case study) is consecutively detected for 5 s, the bio‐logger alters the GPS sampling mode from low to high sampling mode (1 Hz) and keeps the state for at least 3 min. Subsequently, the bio‐logger enters playback standby mode when all 10 recent GPS points are 3D‐fixed and located outside a defined area (at least 1 km away from the colony centre). If the target behaviour (flying) is detected for five consecutive seconds during the playback standby mode, the bio‐loggers start video recording. Video recording continues for about 60 s without the audio playback to record the bird's behaviours before the playback. Then, if the target behaviour persists, the bio‐logger plays back an audio from the speaker and continues video recording. This allows the collection of at least about 60 s of video footage before and after the playback. If the bird stops flying, playback is cancelled, and recording is terminated early to conserve power. To ensure temporal separation between each playback experiment, the bio‐logger was programmed to prevent video recording and playback experiments for a period of 10 min following the completion of a previous playback session. See Text S3 for more details. Note that specific parameters are configurable depending on the research objectives.

### 2.3 Field Experiment

Field experimental data were collected from breeding black‐tailed gulls on Kabushima Island, Japan (40°32 N, 141°33E) between 9th and 23rd May 2024. Permissions were obtained from the Hachinohe City Board of Education (2024‐295) and Aomori Prefecture (2024‐3046). All experimental procedures were approved by the Nagoya University Animal Experiment Committee (V240010‐002). The birds were manually captured at their nests during the incubation period. The bio‐loggers were attached to the birds' feathers on their backs (Figure 2d) using waterproof Tesa tape (tesa SE, Norderstedt, Germany). All 10 birds were male and their body mass ranged from 620 to 735 g. All attached bio‐loggers weighed less than 4.31% of the birds' body mass. The average handling time was 13 min, with a maximum of 19 min. Based on visual observation, we confirmed that the device had little impact on the birds' flight. GPS data confirmed that all tagged individuals embarked on at least one trip. Attempts to recapture the birds were made, starting one or 2 days after deployment, with a maximum attachment period of 4 days. All loggers were successfully retrieved. We prepared two audio files for the field experiments: the call of peregrine falcon ( *Falco peregrinus* ) as a predator and white noise (Text S4 and Figure S2), and one of which was randomly played.

### 2.4 Data Analysis

We used three bird's response indicators to analyse bird behavioural responses to the playbacks. First, vectorial dynamic body acceleration (VeDBA), known as a proxy for energy expenditure (e.g., Wilson et al. 2020), was calculated from tri‐axial acceleration data (25 Hz) for each data point and smoothed over a 2‐s window (S‐VeDBA). S‐VeDBA allowed us to examine how much the intensity of the birds' movements changed. Second, the absolute difference in ground speeds between two successive seconds (AD‐Speed) was calculated based on GPS data (1 Hz) for each data point. AD‐Speed enabled the examination of how much the birds changed their flying speed. Third, we trained an image segmentation model based on YOLOv8 (Jocher et al. 2023), using more than 3000 frames to track the movement of the bird's head and neck in each of the 30 FPS video frames (Figure 1d). We used this segmentation model to create masked images (where the bird's head and neck were masked; Figure S3), and the absolute difference in masked pixel ratios (MPRs) between two successive frames (AD‐MPR) was calculated for each frame. AD‐MPR provided the means to quantitatively examine the birds' reaction in the video data (e.g., vigilance behaviour). See Text S5 for more details.

We defined the period before the playback start timing as the pre‐period and the period during and after the playback as the post‐period (Text S6 and S7 and Figures S4–S7). We then calculated and visualised the average values over 5‐s windows for both the pre‐ and post‐periods for each response indicator (see also Text S8 and Figures S8–S13). Using the difference in the average values (post‐mean—pre‐mean) as response variables, we performed Bayesian statistical modelling to analyse the relationship between the response variables and explanatory variables that may potentially influence the birds' responses: the audio data type (0: noise, 1: predator), location (0: non‐offshore, 1: offshore), body mass (standardised), and the number of playback counts (standardised). Each model assumed that the response variable follows a normal distribution. To account for variation in response to playback across individuals, we included bird identity (individual identification number) as random intercept effects (i.e., the models are hierarchical or mixed‐effects models). Posterior distributions were estimated through Markov chain Monte Carlo (MCMC) sampling using R 4.1.3 (R Core Team 2022) and Stan 2.21.0 (Stan Development Team 2019). We used weakly‐ or non‐informative priors. Bayesian models were run with 4 chains and 8000 iterations, including 6000 warm‐up iterations. We used a thinning rate of 1 and set the adapt delta to 0.99. See Text S9 and Figures S14 and S15 for further details of the Bayesian statistical modelling. To analyse the three response indicators as time‐series data, without aggregating it (e.g., by averaging over fixed time windows), we ran CausalImpact (Brodersen et al. 2015) models without covariates using R 4.1.3 and CausalImpact 1.3.0 (Brodersen et al. 2015). CausalImpact uses Bayesian structural time‐series models to estimate counterfactuals (time‐series data) after an intervention, based on pre‐intervention data. Although the method can incorporate covariates to improve predictions, we used a simple form without covariates—relying solely on the pre‐period data of each of the three response indicators. We compared the observed and the predicted values through visual inspection. See Text S10 for more details of the CausalImpact models. Data pre‐processing was performed in Python 3.11.9 and data visualisation was performed in both Python 3.11.9 and R 4.1.3.

## 3 Results

### 3.1 Summary of Field Experiments

In eight out of 10 field deployments, the bio‐loggers successfully performed autonomous playback experiments at least twice and recorded fine‐scale behaviour data before, during, and after the playback in the wild environment (Table 1). Two of the 10 deployments failed due to problems in the camera and GPS modules, respectively (Text S11). A total of 66 videos were recorded, and the playback experiments were performed in 46 videos. In the other 20 cases, playback experiments were cancelled, and video recordings were terminated because birds were no longer flying when the logger was ready to start playback, preventing unwanted experiments and saving battery consumption. However, in one cancelled case, the program froze and lost control of the camera module, causing it to record videos for more than 40 min (LBP01 S03). The median (range) number of videos and playbacks were 7 (3–15) and 5 (2–12), respectively. The median (range) logging time was 16.7 (7.9–21.3) h. The real‐time on‐board behaviour recognition model was sufficiently accurate for our purposes, with the macro precision, recall, and F1‐score of 0.89, 0.95, and 0.91, respectively. The class precision, recall, and F1‐score for flying were 0.99, 0.91, and 0.95, respectively (Figure S1b). All 46 playback experiments were performed when birds were flying outside the colony as we intended.

**TABLE 1:** Summary of the field experiments. Autonomous (or targeted) playback and behavioural data recording were successfully achieved in 8 out of 10 field deployments.

Test ID	*N* of videos	*N* of playback	*N* of predator	*N* of noise	Playback cancelled	Logging time in hour
LBP00	3	3	2	1	0	21.3
LBP01	4	2	2	0	2	7.9
LBP02	0	0	0	0	0	(1.6)
LBP03	12	9	5	4	3	14.4
LBP04	0	0	0	0	0	(14.3)
LBP05	13	12	3	9	1	13.1
LBP06	5	5	0	5	0	18.5
LBP07	7	5	1	4	2	18.3
LBP08	15	4	1	3	11	15.2
LBP09	7	6	4	2	1	19.5
Total	66	46	18	28	20	128.3 (144.2)

*Note:* Due to logger malfunctions, LBP02 and LBP04 recorded no videos. Their logging durations are shown in parentheses; the total logging time in parentheses includes these. Logging time values are rounded to one decimal place.

### 3.2 Response of the Birds

**FIGURE 3:**
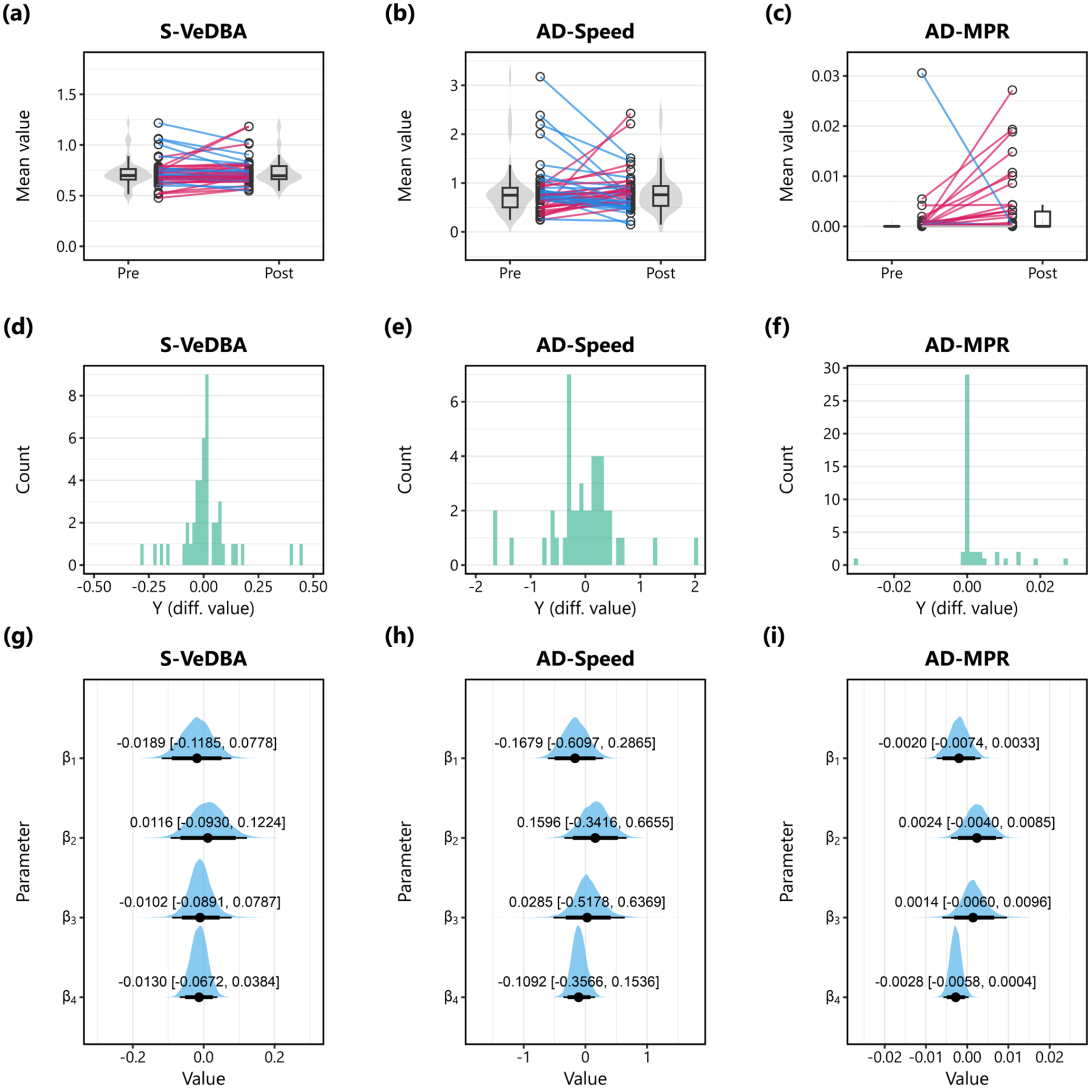
Birds' responses to the audio stimuli based on three response indicators: (a, d, g) the smoothed vectorial dynamic body acceleration (S‐VeDBA; based on 25 Hz tri‐axial acceleration data), (b, e, h) the absolute difference in ground speeds between two successive seconds (AD‐Speed; based on 1 Hz GPS data), and (c, f, i) the absolute difference in masked pixel ratios (MPRs) between two successive frames (AD‐MPR; based on 30 FPS video data). (a, b, c) Slope plots presenting paired comparisons of mean values over 5‐s pre‐ and post‐periods. (d, e, f) Histograms showing distributions of observed difference values (post‐mean—pre‐mean). (g, h, i) Posterior distributions of slope parameters from the models that explored the relationship between the difference values (post‐mean—pre‐mean) and four explanatory variables: audio data type (0: Noise, 1: Predator), location (0: Non‐offshore, 1: Offshore), body mass (standardised), and the number of playback count per audio data (standardised). Black points, thick lines, and thin lines indicate the posterior median, 89% Bayesian credible intervals (BCIs), and 97% BCIs, respectively. Numbers in each plot show the posterior median and 97% BCIs.

**FIGURE 4:**
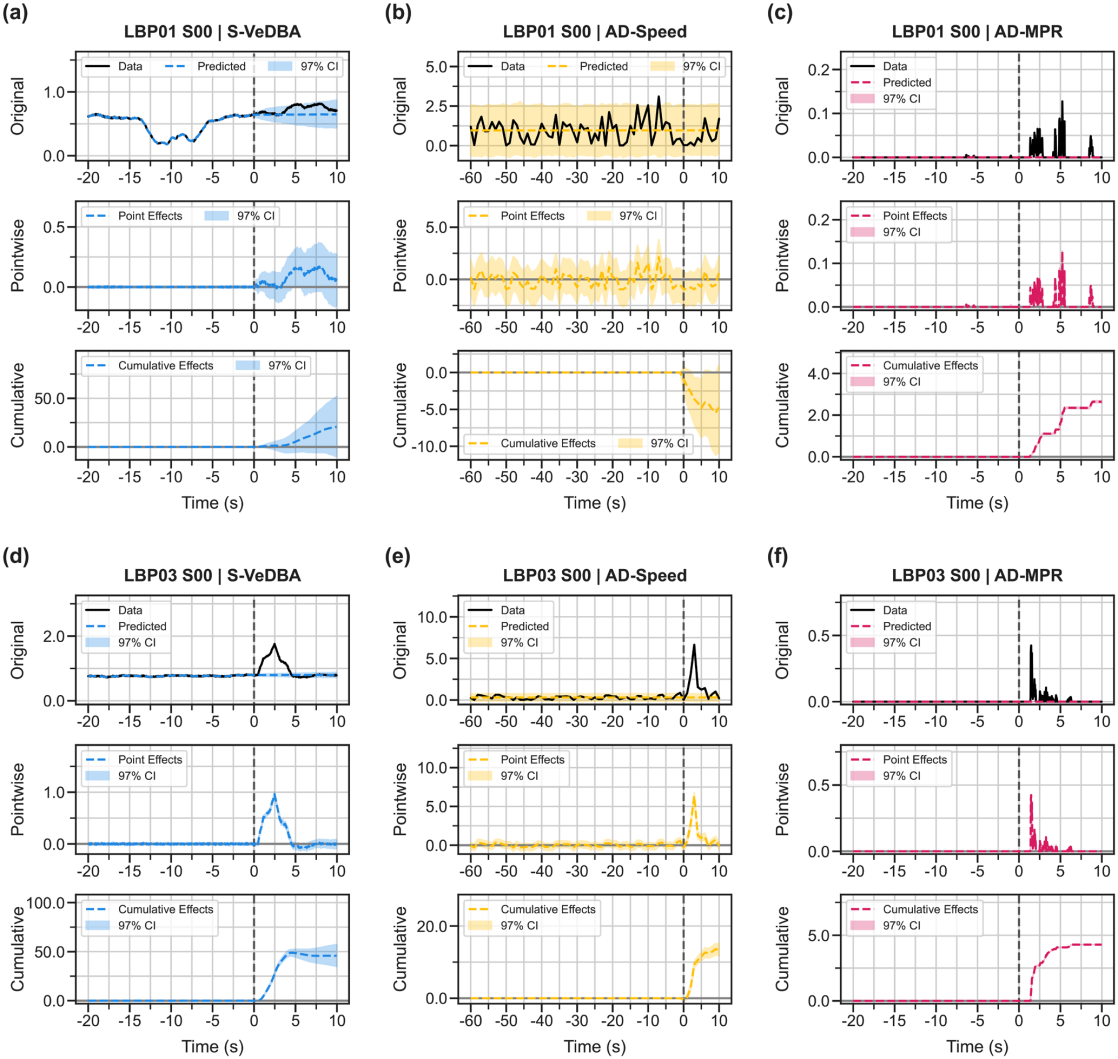
Examples of estimated causal effects of audio playback on time series data for two audio playback sessions. Upper (a, b, c) and lower panels (d, e, f) show the results from LBP01 S00 (predator) and LBP03 S00 (noise), respectively. Each column shows the results of one of the three response indicators: (a, d) the smoothed vectorial dynamic body acceleration (S‐VeDBA; based on 25 Hz tri‐axial acceleration data), (b, e) the absolute difference in ground speeds between two successive seconds (AD‐Speed; based on 1 Hz GPS data), and (c, f) the absolute difference in masked pixel ratios (MPRs) between two successive frames (AD‐MPR; based on 30 FPS video data). In the top panel of each plot, black lines indicate the observed data, while non‐black dashed lines indicate predicted values. In the middle panel of each plot, non‐black dashed lines indicate the point‐wise difference between data and predicted data, while ribbons show 97% Bayesian credible intervals (BCIs). In the bottom panel of each plot, non‐black dashed lines indicate the point‐wise cumulative difference between data and predicted data, while ribbons show 97% BCIs.

Overall effects of audio playback were unclear for S‐VeDBA and AD‐Speed (Figure 3a,b,d,e), but there appeared to be a slight positive effect on AD‐MPR (Figure 3c,f). Although such cases were limited in number across all trials, the birds showed a clear response to the auditory stimuli in some audio playback sessions (both predator and noise sounds; Figure 4; Movies S1 and S2; Text S7). There was no clear relationship between the response variables and three explanatory variables (the audio data type, location, and bird body mass) for any of the three response indicators (Figure 3g–i), while the number of playback counts had a weak negative effect only on AD‐MPR (Figure 3i).

## 4 Discussion

We proposed a system for autonomous playback experiments on bio‐loggers to address the limitations of existing playback methods. As a proof of concept, the field experiments demonstrated that the system successfully performed audio playback at targeted timings and recorded how the birds responded to the playback (but see Text S11 for limitations). Although bio‐loggers using edge computing technology based on machine learning—what we refer to as AI on Animals (AIoA)—have been proposed recently (e.g., Korpela et al. 2020; Tanigaki et al. 2024), to our knowledge, this is the first bio‐logger to perform autonomous audio playback experiments in the natural environment, which could be termed AI playback on animals.

### 4.1 Real‐Time Behaviour Recognition

Real‐time on‐board behaviour recognition using machine learning showed high accuracy in the field experiments. Flying may be an easy behaviour to classify and a simple machine learning model (e.g., Decision Tree) would be sufficient to achieve practical accuracy. Targeting infrequent behaviours, such as foraging, may be more difficult in the case of seabirds because collecting a large amount of data for supervised machine learning is challenging for such behaviours. However, for some diving seabirds, depth sensor data can be used along with acceleration data to help identify foraging behaviour. The rule‐based method using GPS location data also worked, preventing video recording and audio playback at specified locations (i.e., around the colony). It is also possible to record video and play audio only when the logger is located within a specified area. Depending on the research purposes, the bio‐logger can also use GPS or water depth sensor data to estimate the behavioural state of the animals in real time, as Korpela et al. (2020) and Tanigaki et al. (2024) did.

### 4.2 Response of the Birds

In our field experiments, clear responses to audio playbacks, particularly vigilance behaviour as shown in the video data, were observed, but only in a limited number of playback sessions, possibly due to habituation to the audio stimuli, individual variation, or environmental factors, such as wind noise (see Text S12 for more discussion). Also, the birds responded not only to predator calls but also to noise sounds, indicating that they may have been simply startled by the sound from the bio‐loggers on their backs. Playing the sounds from a bio‐logger on the bird's back might have created an unusual scenario, as gulls are more likely to respond to distant predator calls, not those perceived directly from such proximity. Since how the sound is perceived by the subject is critical in playback experiments, regardless of whether fixed speakers or on‐animal playback are used, refining the playback sound or adjusting its volume would be important in future experiments to better mimic a distant predator call. In addition, alternative audio stimuli, such as conspecific alarm calls (MacLean and Bonter 2013), would elicit more ecologically meaningful responses. Nevertheless, the proposed autonomous playback system on bio‐loggers did influence the bird behaviour in several sessions, implying that the system has the potential to manipulate animal behaviour.

### 4.3 Future Development

The proposed system still faces practical challenges, such as the implementation of more effective intervention methods, the development of features to mitigate habituation effects, and the improvements in shape, size, and weight (see also Text S11). Despite the remaining challenges, we believe the proposed system holds potential for future applications in behavioural and ecological studies of various animal species. This is because causal relationships can only be clearly demonstrated through controlled experimental manipulations, and the proposed system is expected to facilitate intervention studies in natural environments across a wide range of animal taxa. The system can be extended to other intervention modalities in the future, further increasing its versatility. For example, it may be possible to change the magnetic field around the animals. Magnetic fields are known to play an important role in the navigation of diverse animal species (Mouritsen 2018) and the proposed system could therefore be used to test a variety of important hypotheses in the natural environment, without capturing and transporting animals to a laboratory. Another exciting and challenging future direction is to develop a system that incorporates a feedback loop using recorded data to flexibly or interactively (King 2015) modify the intervention timing, method, and/or its parameters (e.g., to avoid habituation to stimuli (Flower et al. 2014)). Such flexible systems may be relatively easily achieved in not fully open environments (e.g., Nourizonoz et al. 2020) but in natural environments. If achieved, it would greatly expand the possibilities of bio‐logging studies and may also help mitigate human–wildlife conflicts by enabling targeted, non‐lethal behavioural interventions, such as deterring wild animals that encroach upon human‐inhabited areas.

## Author Contributions

**Ryoma Otsuka:** data curation (supporting), formal analysis (lead), methodology (equal), software (lead), validation (lead), visualization (lead), writing – original draft (lead), writing – review and editing (equal). **Hibiki Sugiyama:** data curation (equal), methodology (supporting), writing – review and editing (equal). **Yuichi Mizutani:** data curation (equal), methodology (supporting), writing – review and editing (equal). **Ken Yoda:** conceptualization (equal), data curation (equal), funding acquisition (equal), methodology (supporting), writing – review and editing (equal). **Takuya Maekawa:** conceptualization (equal), formal analysis (equal), funding acquisition (equal), methodology (equal), project administration (lead), software (equal), supervision (lead), writing – review and editing (equal).

## Disclosure

Statement of Inclusion: We conducted our research in Japan and all authors were born and raised in Japan. None of the authors is from Hachinohe city, where we conducted the field experiment; however, the research team at Nagoya University, led by Ken Yoda, has built strong and collaborative relationships with local communities over the years.

## Conflicts of Interest

The authors declare no conflicts of interest.

## Supporting information


**Video S1.** Example of a playback session (LBP01 S00; predator call) using the proposed autonomous playback system implemented in bio‐loggers. Synchronised video footage, acceleration, and ground speed data are shown for 20 s before and after the playback onset.

**Video S2.** Example of a playback session (LBP03 S00; white noise sound) using the proposed autonomous playback system implemented in bio‐loggers. Synchronised video footage, acceleration, and ground speed data are shown for 20 s before and after the playback onset.

Appendix S1.

## Data Availability

The data and source code from this study are available from https://github.com/ryoma‐otsuka/logbot‐playback‐2024.
