# Supplementary material for: Real‐Time Behaviour Recognition on Bio‐Loggers Enables Autonomous Audio Playback Experiments in Free‐Ranging Seabirds
Source: Ecol Evol. 2025 Aug 6;15(8):e71832. doi: 10.1002/ece3.71832 (PMC12326428; doi:10.1002/ece3.71832)
Supplement: Supplementary file 3 — Appendix S1. [file ECE3-15-e71832-s002.pdf]

Supporting Information for

Real-time behaviour recognition on bio-loggers enables autonomous audio playback experiments in free-ranging seabirds

Ryoma Otsuka[1, 2], **Hibiki Sugiyama**[2], **Yuichi Mizutani**[2], **Ken Yoda**[2], **Takuya Maekawa**[1, 3]

[1] Graduate School of Information Science and Technology, The University of Osaka, Suita, Osaka 565-0871, Japan.
[2] Graduate School of Environmental Studies, Nagoya University, Nagoya, Aichi 464-8601, Japan.
[3] Institute for Advanced Co-Creation Studies, The University of Osaka, Osaka 565-0871, Japan.

✉ ryoma.otsuka87@gmail.com (First author | Ryoma Otsuka)
✉ maekawa@ist.osaka-u.ac.jp (Corresponding author | Takuya Maekawa)

Last updated: July 2, 2025

Table of Contents

Supplementary Text 3

Supplementary Materials and Methods 3

Text S1. Details of waterproofing methods 3

Text S2. Training and implementation of the behaviour recognition model 3

Text S3. Details of audio playback systems on bio-loggers 4

Text S4. Preparation of audio data 4

Text S5. Three response indicators 5

Text S6. Deciding the boundary between pre- and post-period 6

Text S7. The time gap between frames and audio data in videos 10

Text S8. Analysis of behavioural changes after audio playback 11

Text S9. Bayesian statistical modelling 12

Text S10. Causal inference using a CausalImpact model 13

Supplementary Results and Discussion 14

Text S11. Limitations and future development of the bio-logger 14

Text S12. Further results and discussion on the birds' responses to audio playbacks 15

Supplementary Data 19

Supplementary Figures 19

Figure S1 19

Figure S2 20

Figure S3 21

Figure S4 22

Figure S5 23

Figure S6 24

Figure S7 25

Figure S8 26

Figure S9 27

Figure S10 28

Figure S11 29

Figure S12 30

Figure S13 31

Figure S14 32

Figure S15 33

Supplementary Text

Supplementary Materials and Methods

Text S1. Details of waterproofing methods

The main body of the bio-logger was waterproofed with resin. The front of the camera was fitted with an acrylic plate, fixed with glues and resin (Figure 2c). To create some space in front of the speaker while waterproofing, a cork with a hole (a few millimetres thick) was stuck to the front of the speaker and a waterproof cloth was wrapped around it and fixed with glue and resin (Figure 2c). In the same way, the top of the microphone hole was not sealed with resin but covered with a waterproof cloth or tape over a cork, which was fixed with glue (Figure 2a). We used a liquid gasket to waterproof the 4-pin terminal and its surrounding area for several to 12 hours before attaching the bio-loggers to the birds. The bio-logger weights reported in the text were measured after completing all the waterproofing processes mentioned above.

Text S2. Training and implementation of the behaviour recognition model

A Decision Tree model was developed to classify the ”flying” and ”non-flying” behaviours of black-tailed gulls. The model training and testing were performed using Python 3.11.9 and scikit-learn 1.4.2. We used acceleration data that were previously collected from black-tailed gulls during 2018 and 2019, and tested on data collected in 2022. The acceleration data were labelled using the video footage collected by our bio-loggers. To reduce the influence of differences in attachment positions and sensor devices, the sum of squares was calculated from tri-axial raw acceleration data for each data point. The one-second windows were extracted from the labelled segments without overlap. Subsequently, four features (mean, variance, mean cross, and kurtosis) were calculated for each window using a vector of the sums of squares. The four features and a label for each window were used for training and testing. The test results are as follows: the macro precision, recall and F1-score were 0.87, 0.95 and 0.90, respectively, while the class precision, recall and F1-score for flying behaviour were 0.99, 0.92 and 0.95, respectively (see Figure S1a for the confusion matrix). In addition, we evaluated the performance of real-time behaviour recognition by comparing the prediction results recorded every second with the collected video footage (ground truth). The simple Decision Tree model demonstrated sufficient performance in the binary classification of flying and non-flying behaviour (see the Results of the main text & Figure S1b). To enable on-board processing, the Decision Tree model was converted from Python to C++ code and implemented on the bio-logger. Decision Tree models can be represented by simple conditional statements, thus easily implemented in the bio-logger. During data logging, the sum of squares and the above-mentioned four features (mean, variance, mean cross, and kurtosis) were calculated from tri-axial raw acceleration data and the implemented Decision Tree model returned prediction output (flying or non-flying) based on the features every second.

Text S3. Details of audio playback systems on bio-loggers

When the bio-logger is in the playback standby mode, and if a target behaviour is detected for five consecutive seconds, the bio-loggers start video recording. It takes approximately 2–3 seconds for the camera module to start recording after receiving a command. Video recording continues for 60 seconds without the audio playback to record the bird's behaviours before the playback. Then, over the subsequent 65 seconds, the bio-logger examines the last 10 seconds of behaviour recognition results. If the target behaviour is recognised for a minimum of six seconds out of 10 and the most recent behaviour recognition result is a target behaviour, the bio-logger randomly plays audio from the speaker. After the playback, the bio-logger continues to record video until the total duration of this recording session reaches 185 seconds, ending up 182–183 s length of video data (60 + 65 + 60 s, with 2–3 s subtracted). If the playback experiment was not conducted during the above 65 seconds, the bio-logger terminates video recording, ending up 123–124 s length of video data (60 + 65 + 1 s, with 2–3 s subtracted).

The playback standby mode was programmed to last for 30 minutes as a default and extended by 30 minutes each time the bio-logger recorded video data. However, black-tailed gulls may land in the land or sea near their colony to rest or bathe after flying away from the colony or resting sites. In such cases, the bio-logger can automatically abort the GPS high sampling mode (1 Hz) and go back to the default, low sampling mode to conserve battery power. After the first 3 minutes and a further 10 minutes have elapsed since the GPS sampling rate was changed to 1 Hz, the system starts checking the results of behaviour recognition every minute. If, for five consecutive minutes, the target behaviour (flight) was only recognised for less than 10 seconds in the last minute, the GPS sampling rate returns to the default, low sampling rate mode. In the low sampling rate mode, the bio-logger activates the GPS module for only 2 minutes every 15 minutes, ensuring the acquisition of GPS location data every 15 minutes.

Text S4. Preparation of audio data

We prepared two audio files for the playback experiments in the filed: peregrine falcon (*Falco peregrinus*) call and white noise (Figure S2). Peregrine falcon is occasionally observed in and around Kabushima Island, and it is one of the potential predators of black-tailed gulls in Kabushima Island (see [17]). Both audio files are adjusted to 4.2 seconds in length. The sampling rate of these audio files was 8,000. We calculated root mean square (RMS) from the falcon's call data, employing a frame length of 200 and hop length of 100, and adjusted the white noise data so that the average RMS of the two audio files was approximately the same value (= 0.21334). The predator call data was sampled from an online video platform and processed (noise reduction and amplification) using Audacity 3.2.5 (http://www.audacityteam.org/; https://github.com/audacity/audacity). The noise audio file was created using Python 3.11.9, scipy 1.13.0 and librosa 0.9.1. We measured the sound levels of actual sound played by the bio-logger's speaker using the sound level meter (CHE-SD1, SANWA SUPPLY, Okayama, Japan) 10 times (five times per device) per audio file type using two devices. We measured the sound levels at a 15 cm distance outside because the distance between the bird's ears and the bio-logger attached to its back was approximately $<$15 cm. The median (range) sound levels of predator call and white noise at 15 cm distance were 85.2 (80.7–89.9) and 79.3 (74.2–84.0), respectively. For the test ID LBP00–LBP07, the bio-loggers were programmed to select

an audio file ("predator call" or "white noise") just before playing the audio from the speaker based on the time (second) when all criteria are met for audio playback. The bio-logger played "predator call" if the second of the time was an odd number (e.g., 3 or 59), while the bio-logger played "white noise" if the second of the time was an even number (e.g., 6 or 48). For the test id LBP08 and LBP09, the bio-loggers were programmed to randomly select an audio file using Arduino's built-in "random" function.

Text S5. Three response indicators

To quantitatively analyse birds' response to the playback, we designed and used the three response indicators based on acceleration, GPS, and video data.

Response indicator 1. S-VeDBA based on Acceleration data

Raw triaxial acceleration data were sampled at 25 Hz throughout data logging. We first calculated static components by calculating the 3 seconds moving average [14] and obtained dynamic components by subtracting static components from the raw data for each axis. Then, we calculated the square root of the sum of squares of the three axes data (L2 norm). This feature is known as vectorial dynamic body acceleration (VeDBA) which is regarded as a proxy for energy expenditure [11, 18]. We obtained smoothed VeDBA by calculating the moving average over 2 seconds; we refer to this as S-VeDBA in this study. The calculated moving average value was assigned to the rightmost index of the 2-second-length window to ensure that the future values would not affect the moving average value at that index. Otherwise, the waveform of the acceleration data corresponding to the bird's movement after the start of the audio playback would affect the data points recorded before the playback. We used this as the first response indicator because we predicted that the intensity of the birds' movements might increase (e.g., birds may exhibit intense flapping), and their energy expenditure would also increase in response to the playback.

Response indicator 2. AD-Speed based on GPS data

Fine-scale trajectories of the birds before, during and after the playback were recorded as GPS location data at 1 Hz. From the latitudes and longitudes, we calculated a travel distance (m) in 2D space for each second, which is equivalent to the ground speed (m/s). Then, we calculated the absolute value of the difference between travel distances of two successive seconds (those of time steps t-1 and t), which is referred to as AD-Speed in this study. We used this as the second response indicator because we predicted that the birds might change their flying speeds in response to the playback.

Response indicator 3. AD-MPR based on video data

The video data were recorded at 30 FPS before, during and after the playback. When a bird is flying (flapping flight or grinding), frames of our video data usually show only the sky and the sea or land (Figure S3). However, the head and neck of the bird are often in the frame when the bird is stretching the neck or moving the head for vigilance, swooping, soaring or changing direction rapidly. In such cases, the amount of change in the position and area of the head and neck within a frame tends to be large between successive frames. For some deployments, the head or neck of the bird was almost always in the frames, but its

position and size did not vary significantly between successive frames when the birds were stably flying. Therefore, we thought that the birds' response in the video data could be quantitatively analysed by measuring the changes.

To detect and segment the head and neck of the bird in each frame, we trained the image segmentation model, YOLOv8 [6] using a custom dataset. To create the custom dataset, we extracted and annotated a total of 3,245 frames from 66 videos, with 1,812 images including a bird label. For model training, we split the dataset into 90% train, 9% validation and 1% test dataset. The annotation, data split, data preprocessing (auto-orientation and resizing data to 640 by 640), and data augmentation (3×) of training data were performed using Roboflow [3]. Data augmentation methods included horizontal flip, random crop (between 0 and 12 per cent of the image), random rotation (between -10 and 10 degrees), random brightness adjustment (between -15 and 15 per cent), random exposure adjustment (between -10 and 10), random gaussian blur (between 0 and 1.5 pixels), and noise (0.1 per cent of pixels). We first initialised the YOLOv8 segmentation model (Jocher et al., 2023) with pre-trained weights (yolov8s-seg.pt). Then we trained the model on our custom dataset using Python 3.10.12, PyTorch 2.3.0, ultralytics 8.0.196, Docker 24.0.7 and cuda 11.8.0 on a Linux server (Ubuntu 22.04 LTS) with two GPU NVIDIA RTX A6000 (48GB). The maximum number of training epochs was 300 and the patience parameter was set to 50 to avoid overfitting; the actual training stopped after 130 epochs. The image size of training data was 640 by 640 with 3 channels and the batch size was 16. The mAP50 (mAP50-95) for validation and test datasets were 0.98 (0.89) and 0.99 (0.90), respectively. Using the custom-trained YOLOv8 segmentation model, we ran predictions on all extracted frames (640 by 480 with 3 channels), and this created a masked image for each frame. Although these extracted frames included annotated images in the custom dataset used for model training, data leakage problems and overfitting are not that much of a concern in this method. This is because the goal of our image segmentation task was to efficiently and precisely predict the location and area of the bird in video frames and these video frames included those used for model training and those are very similar to images used for model training. We then counted the number of masked pixels for each frame (Figure S3bc). Masked pixel counts were then converted into masked pixel ratio (MPR) by dividing the number of total pixels. Then the absolute difference in masked pixel ratios (MPRs) between two successive frames (we refer to this as AD-MPR in this study) was calculated for each frame.

Text S6. Deciding the boundary between pre- and post-period

We defined the period before the playback start timing as pre-period, and the period during and after the playback as post-period. To analyse fine-scale birds' movements in response to the audio playback, it is crucial to estimate the timing of when the audio playback started (i.e. the boundary between pre- and post-periods) as precisely as possible. The required accuracy level varies depending on the analysis goals and method used. For example, if the goal is to estimate the reaction time, such as how many milliseconds after hearing the audio stimuli the bird starts to react, very high accuracy (e.g., $<$10 ms) is required. On the other hand, if the goal is to examine whether there was a difference in behaviour during the 5–10 seconds before and after the audio playback, an error of more than one second is unlikely to significantly impact the results of the analysis. Yet, in any case, the estimated audio playback start timing should be earlier than or

equal to the true audio playback start timing.

The goal of our analysis is to evaluate whether there was a change in the bird's behaviour between pre- and post-periods. Therefore, our analysis does not require very high accuracy. The accuracy level of less than 1–2 seconds would be sufficient for our data analysis as long as the estimated playback start timing is earlier than or equal to the true audio playback start timing. To better understand how the time difference between the true playback start timing and the playback start timing recorded in the logging data would affect the results of our data analysis, we performed post-hoc validation experiments. We describe the methods, results and interpretation of the post hoc validation experiments in the following subsections. In conclusion, we decided to use the playback start timing recorded in the logging data without any adjustments for acceleration and GPS data, while we decided to adjust the playback start timing for video data based on a difference between expected and recorded numbers of frames. We recognise that these approaches do not perfectly synchronise the timing of each modality's data with the true audio playback start timing, and there might be slight timing discrepancies between modalities. Therefore, we do not discuss response time with high precision or compare response time between modalities, rather we simply focus on whether there was a change in each response indicator after the audio playback start timing.

Logging data

We first describe how the data were recorded in our bio-loggers. Figure S4 illustrates a schematic overview of our Arduino (C/C++) program. Each loop is 40 ms long, and 25 loops (the loop index = 0, 1, 2, . . . , 24) correspond to 1 second of data. We programmed the bio-logger in this way because we could not control the FIFO functions of the IMU (BMI270). All the data recorded within 1-second long 25 loops are copied in the 25th loop (the loop index = 24) and saved to the SD card as binary data in the next 7th loop (the loop index = 6). The data recorded within 1-second long 25 loops include those recorded at 1 Hz (e.g., RTC, GPS, magnetometer) and 25 Hz (e.g., accelerometer, gyroscope).

After downloading all the binary data from the SD card of the bio-logger, our parsing program generates CSV files based on the binary data. In the CSV file (logdata.csv), each row corresponds to a single timestamp (0, 40, 80, . . . , 960 ms) of the 25 Hz data while data recorded at 1 Hz were written in the first row of any second (i.e. 0 ms row). The CSV file contains a column indicating whether the audio playback was ongoing at the first row of each second (0 ms), recorded as a binary variable (0 or 1). Thus, the start timing of the audio playback is recorded with a precision of <1 second. Similarly, the CSV file also contains a column indicating whether the camera was recording video or not. The video recording start command was sent in the 3rd loop (the loop index = 2), while the audio playback command was sent in the 4th loop (the loop index = 3). This means that an audio playback start timing recorded in the CSV file is always earlier than the true audio playback start timing, conceptually.

Delay time after sending audio playback command

Although we know roughly when the audio playback command was sent, we also need to consider the time between the timing of sending the playback command and the timing of the actual sound being produced by the speaker (i.e. delay time). To estimate the delay time, we performed a post hoc experiment. We prepared

a program for this experiment by modifying the program used for the field experiment. The modified program repeatedly records a video of 17–18 seconds every 30 seconds (the duration from the sending recording start command to sending the recording stop command was set to approximately 20 seconds). During the video recording, the audio playback command was programmed to be sent about 10 seconds after sending the video recording start command. A human experimenter (the first author) continuously touched the bio-logger placed on the desk during the experiment. When the audio was not coming from the speaker, the bio-logger was placed with its side facing down. When the sound began to play and until the end of the audio playback, the bio-logger was placed with its bottom facing down. The experimenter tried to change the orientation of the bio-logger as quickly as possible after the sound started or stopped. We repeated the experiment 11 and 8 times for two different devices, respectively.

This post-hoc experiment allowed us to calculate the time (in milliseconds) between the audio playback start timing recorded in the logging data (at 0 ms) and when the accelerometer values changed. We denote this time as $T_{total\_response}$ and it can be formulated as the sum of the following components as below:

$$T_{total\_response} = T_{command} + T_{delay} + T_{human\_auditory\_response} + T_{acc\_response} \quad (1)$$

, where $T_{command}$ is the time from the start of a 1-second long 25 loops (i.e. 0 ms) to the loop where the audio playback command is sent, T_{delay} is the time between sending the audio playback command and the actual sound being broadcasted from the speaker, $T_{human\_auditory\_response}$ is the reaction time to auditory stimuli, and $T_{acc\_response}$ is the time between moving the bio-logger and the accelerometer responding. In our program, $T_{command}$ should be approximately 120 ms. The average reaction time to auditory stimuli by humans (healthy medical students) is about 228 ms [5] and we used this value as $T_{human\_auditory\_response}$. We assumed that the $T_{acc\_response}$ was negligibly small and considered it as zero. Therefore, the following equation can estimate T_{delay}.

$$T_{delay} = T_{total\_response} - 338 \quad (2)$$

We estimated the $T_{total\_response}$ using the post hoc validation experiment data and calculated the T_{delay}. The median (range) value was 442.00 (302.00–582.00) (Figure S5). This experiment also confirmed that an audio playback start timing recorded in the CSV file is always earlier than the true audio playback start timing.

Acceleration data

Although it is important to recognise that there are certain amounts of delay time and it may fluctuate, this delay time would not affect the results of our data analysis on acceleration data. This is because 1) we are simply interested in whether there were differences in the bird's behavioural indicators between the pre- and post-periods, 2) the estimated delay time is less than 1 second while our analysis uses a time window of 5 seconds or more, and 3) the audio playback start timing recorded in the CSV file (logdata.csv) is earlier than the true audio playback start timing. Therefore, we determined that it is not necessary to adjust the timestamps of the acceleration data for our data analysis purposes.

GPS data

The GPS data before, during and after the playback were recorded at 1 Hz and the GPS data were obtained in the 23rd loop (the loop index = 22). This means that GPS data recorded at time t (playback start timing row) in the logdata.csv file are the data parsed approximately 760 ms (40×19) after the 4th loop (the loop index = 3) in which the audio playback start command was sent. Therefore, we also determined that it is not necessary to adjust the timestamps of the GPS data for our data analysis purposes.

Video data

We also need to examine the time discrepancy between the video frame data and the audio playback start timing. This is because it takes several seconds for the camera module to capture the first image after receiving the command. In other words, there is a delay of several seconds before the actual video recording starts after sending the command, and the delay time would vary. To account for the delay time after sending the command, we adjusted the playback start timing for the video frame data using the difference between the expected number of recorded frames and the actual number of recorded frames. The relationship between the expected number of frames (F_{exp}), the actual number of frames (F_{actual}), and the number of frames corresponding to the delay time after the video recording start/stop command is issued ($F_{start\_delay}/F_{stop\_delay}$) can be formulated as below.

$$F_{exp} - F_{actual} = F_{start\_delay} - F_{stop\_delay} \tag{3}$$

First, we simply determined the actual number of recorded frames as the total number of frames extracted from each video data (AVI file). Next, we considered the expected number of frames, which is the number of frames that should have been recorded assuming no delay time occurred after the command was issued. In our Arduino program (Figure S4), the video recording start command is sent in the 3rd loop (the loop index = 2), while the video recording stop command is sent in the 6th loop (the loop index = 5). The camera status variable (or column) in the CSV file named "camera_recording" will be True (from False to True) immediately after the video recording start command is sent, while it will be False (from True to False) immediately after the video recording stop command is sent. Therefore, the video recording start command is issued approximately 80 ms after a certain time t (.000 s), at which the "camera_recording" variable changes from False to True in the CSV file. The video recording stop command is issued approximately 200 ms after the time t + camera_recording_time (e.g., 185.000 s), at which the "camera_recording" variable changes from True to False in the CSV file. Strictly speaking, there is likely some variation in the timing of the video recording start/stop commands being issued, but this variation is small compared to the delay time that occurs after the commands are issued. When a 30 FPS video is recorded for 185.000 seconds, 5,550 frames are expected to be recorded. Based on the relationship between the timing of the video recording start/stop commands and the "camera_recording" variable mentioned above, the actual recording duration is estimated to be 185.120 seconds. This means that 5,550 + 3.6 = 5,553.6 (rounded to 5,553) frames are expected to be recorded. If the audio playback was cancelled, the video recording was stopped after 126 seconds, and the expected number of frames would be 3,783 ($126 \times 30 + 3$). We calculated the difference in the number of frames between the expected (5,553 or 3,783) and actual numbers of frames for each of

the 64 videos. One very long video (LBP01 S03) and one video that stopped at approximately 2 minutes and 24 seconds due to low battery (LBP06 S04) were removed from this analysis. The median (range) was 78 (55–87) frames (Figure S6).

The delay times (and the corresponding number of frames) after issuing video recording start (F_{start_delay}) and stop (F_{stop_delay}) commands are not zero, but the former is much longer than the later. This is because starting video recording takes more time than stopping video recording. When starting video recording, it begins capturing the first frame after some preparation processes, such as turning on the SD card and initializing the camera module. However, when video recording is stopped, capturing an image is stopped first, followed by any other termination processes, such as writing the last image to the SD card and turning off the SD card. Therefore, F_{stop_delay} is negligibly small (e.g., several frames) compared to F_{start_delay} and we can regard it as zero for simplicity. In this case, F_{start_delay} can be calculated as below.

$$F_{start\_delay} = F_{exp} - F_{actual} \tag{4}$$

We calculated this value and used the value to adjust the timestamps for each video data.

We recognise that the adjusted timestamps may not be very accurate (e.g., $<$10 ms accuracy) due to various factors such as time drift for processing each frame. However, as with GPS and acceleration data, the purpose of our data analysis is to determine whether there were any behavioural changes before (pre) and during/after (post) the audio playback. Therefore, high precision ($<$1 second) is not required. As long as the audio playback start timing used for analysis does not occur after the true timing, a time lag of 1–2 seconds will not significantly affect the results (and their interpretation) when looking at the difference in average values over 5 (or 10, 15, 20) seconds or when using CausalImpact analysis (again, note that we did not analyse reaction time after the audio playback start timing).

Text S7. The time gap between frames and audio data in videos

Our bio-logger had a microphone, and it recorded sound data which were integrated into the video data. The sound data recorded by this microphone allowed us to confirm that the bio-logger had completed the audio playback. However, We found that in some videos where the birds responded to the audio stimuli, the birds appeared to start moving slightly before the sound played. This led us to realise that there was a short time gap ($<$1 second) between the recorded video and sound data. We found that the short time gap is due to the nature of the software used to convert the recorded images (frames) and sound data to movie data (AVI format). This short time gap can be adjusted post hoc either manually or systematically. Note that the sound data in Movies S1 & S2 have been manually adjusted to synchronise with the timing of the video data based on the results of the following experiment.

To achieve accurate timing adjustment, we performed the following handclap experiment and estimated the time gap. When connected to a PC, our bio-logger can start and stop video recording at any desired time through operations performed on the PC (e.g., by clicking the REC button in our GUI application). In this post hoc experiment, we record videos using this function and the same video settings used in the field experiment (i.e. 30FPS, 640×480). During the recording of each video footage, a human experimenter

(the first author) clapped hands 5 times, with each handclap being separated by at least approximately 1 second. The handclaps were made at a distance of approximately 500 mm from the front of the bio-logger's camera, and the distance from the front of the camera to the microphone was approximately 19 mm. We used 3 devices and conducted the recording experiments 3 times per device, resulting in a total of 45 handclaps (15 handclaps per device).

We visually examined each frame extracted from the videos. The first frame in each handclapping session where both hands are together was identified and referred to as the "handclap start frame". The sound data were extracted from video data (AVI files) using Audacity 3.4.2 (http://www.audacityteam.org/; https://github.com/audacity/audacity) and exported as WAV files. Then, we loaded and normalised the sound data and identified handclap timing (as indices) using a handclap detection algorithm. The sampling rate of the sound data was 32,000 Hz. For example, if the 91st frame is identified as the handclap start frame, the elapsed time from the start of the video recording to the handclap (as identified from the video data) is approximately 3,000 ($= 90/30 \times 1,000$) ms. If the identified index of a handclap is 100,001, the elapsed time from the start of video recording to the handclap (identified from the sound data) is approximately 3,125 ($= 100,000/32,000 \times 1,000$) ms. In this case, the gap time between video frames and sound data is approximately 125 ms. We calculated the gap time for all handclap sessions and the median (range) was 221.45 (185.91–247.49) ms (Figure S7). These gaps are roughly equivalent to 5 to 8 frames of 30 FPS video data.

Text S8. Analysis of behavioural changes after audio playback

Our playback experiments were randomised, and the intervention effect of playback experiment i can be simply formulated as below:

$$Y_i = Y_{post[i]} \; - \; Y_{pre[i]} \tag{5}$$

, where $Y_{pre[i]}/Y_{post[i]}$ is the mean value of one of the three response indicators during a certain second (e.g., 5, 10, 15, or 20 s) of pre/post-periods of the audio playback start timing. We calculated $Y_{[i]}$ for each playback session and for the four different time windows (5, 10, 15 and 20 s) and visualised them as Figure S8. In the Results and Figure 3 of the main text, we showed the results using a 5-second time window. Considering that the length of the time window used for analysis may influence the results, we also visualised the data using time windows of 10, 15, and 20 seconds.

In addition, we calculated the mean intervention effect as below.

$$E = \frac{1}{N} \sum_{i=1}^{N} (Y_{post[i]} \; - \; Y_{pre[i]}) \tag{6}$$

We calculated E for both predator and noise playback for the three response indicators and the four different time windows (5, 10, 15, and 20 s) and visualised them in Figure S9.

Moreover, we hypothesised that the degree of response may be related to the number of playbacks. If a bird showed some responses, clearer and stronger responses would be observed in the earlier playbacks, while

responses would diminish or become smaller in the later playbacks. This effect is referred to as the habituation effect which has been reported in audio playback experiments (e.g., [4]. To visually inspect the relationship, we plotted the number of playbacks for both the predator call and noise sound on the horizontal axis and $Y_{[i]}$ for one of the three response indicators on the vertical axis. Figure S10, S11, S12, and S13 illustrate the results with 5-, 10-, 15-, and 20-second time windows, respectively. We also examined this relationship through Bayesian statistical modelling which we describe below.

Text S9. Bayesian statistical modelling

We performed Bayesian statistical modelling to analyse the relationship between the changes in response indicators and covariates that may have an impact on them. All analysis described in this section was performed using R 4.1.3 [12], Stan 2.21.0 [15], and RStan 2.21.7 [16]. We checked the convergence of MCMC sampling by visually inspecting trace plots. We also checked that Rhat values of all parameters were less than 1.01.

We ran linear mixed models to analyse the relationship between the response variable, $Y_{[i]}$ (for each response indicator), and the following four variables: audio file (0: noise, 1: predator), location (0: non-offshore, 1: offshore), body mass (standardised), and the number of playback counts per audio file (standardised). The model structure is formulated as below.

$$u_{[k]} \sim Normal\,(0, \sigma_u) \tag{7}$$

$$Y_{[i]} \sim Normal(\mu_{[i]}, \sigma_Y) \tag{8}$$

$$\mu_{[i]} = (X\boldsymbol{\beta})_{[i]} + \alpha + u_{[group[i]]} \tag{9}$$

The parameter α is an intercept parameter. The parameter $u_{[k]}$ is the random effect (random intercept) for each deployment (i.e. bird), and we assumed that $u_{[k]}$ follows a normal distribution with a mean of 0 and a standard deviation of σ_u. We assumed that $Y_{[i]}$ follows a normal distribution with a mean of $\mu_{[i]}$ and a standard deviation of σ_Y. The $\boldsymbol{\beta}$ is a vector of slope parameters and X is a design matrix ($N \times 4$ matrix of explanatory variables, where N is the sample size). We used the following weakly- or non-informative priors.

$$\boldsymbol{\beta} \sim Normal\,(0, 5) \tag{10}$$

$$\alpha \sim Normal\,(0, 5) \tag{11}$$

$$\sigma_u \sim Normal\,(0, 3) \tag{12}$$

$$\sigma_Y \sim Normal\,(0, 3) \tag{13}$$

The hyperparameters for MCMC sampling were as follows: the number of chains was 4, the number of iterations was 8,000, the number of warmups was 6,000, the thinning rate was 1, and the adapt delta was 0.99. Using MCMC samples, we calculated posterior median, 89%, and 97% Bayesian credible intervals (BCIs) of $\boldsymbol{\beta}$. The posterior distributions as well as the posterior median, 89%, and 97% BCI values of $\boldsymbol{\beta}$ are

shown in Figure 3 in the main text. The posterior distributions of parameters α, $u_{[k]}$, and σ_u are shown in Figure S14.

We recognise that the probability density distributions of the observed data have special shapes (i.e. they are not likely to be normal distributions), as shown in the histograms in the main text (Figure 3def) and the black lines in Figure S15abc. In particular, in the case of AD-MPR, there is a characteristic where if the bird head is not visible in any of the video frames and there is no clear response to the playback, the data values in both the pre-period and post-period become zero, resulting in a difference of zero. Therefore, there is a discrepancy between the probability density distribution estimated from such observed data and the posterior predictive distribution from the models that assumed a normal distribution of each response variable (Figure S15abc). To understand how the choice of probability distribution would influence the results, we also ran a model using a Student-t distribution (with 2.2 degrees of freedom) instead of a normal distribution. For these models, we used the same priors for α and $\boldsymbol{\beta}$, but used the following priors for σ_u and σ_Y.

$$\sigma_u \sim Normal\,(0, 10) \tag{14}$$

$$\sigma_Y \sim Normal\,(0, 10) \tag{15}$$

We observed that the posterior predictive distribution in this case was less affected by “outliers” and was more similar to the probability density distribution of the observed data (Figure S15def). However, we would like to analyse the influence of “outliers” that tend to be ignored by the model using the Student-t distribution. Therefore, we decided to use a normal distribution.

Text S10. Causal inference using a CausalImpact model

To analyse the temporal changes in the above three indicators before (pre) and during/after (post) the playback, we employed the CausalImpact [1] approach. We used R 4.1.3 and CausalImpact 1.13.0 on a laptop (Windows 11 Pro) to perform the CausalImpact analysis. Briefly, CausalImpact employs Bayesian structural time series models to estimate time-series data after the intervention point, i.e. counterfactual, using time-series data before the intervention point. The models can utilise covariants X, which are expected to help predict counterfactuals. When the covariants X are not provided, the CausalImpact only utilise the data sequences before the intervention point (it builds a local-level model). We used this simple approach without covariates in this study. We set the pre- and post-durations in seconds (and data points) as below: 20 and 10 seconds (500 and 250 data points) for acceleration data-based indicator (S-VeDBA), 60 and 11 seconds (60 and 11 data points) for GPS data-based indicator (AD-Speed), and 20 and 10 seconds (600 and 300 data points) for video data-based indicator (AD-MPR). In the case of AD-MPR, there were cases where all the pre-period data values were zero. In such cases, the CausalImpact model could not run properly. Therefore, to facilitate the analysis, we added a very small value (1e-7) to a randomly sampled index from the pre-period data. We set the alpha parameter to 0.03 and we reported and visualised the mean and 97% credible intervals (CI) to roughly summarise the posterior distributions (Figure 4).

Supplementary Results and Discussion

Text S11. Limitations and future development of the bio-logger

One of the limitations of the proposed system is the short battery life, which is a common drawback of the bio-logger with video cameras [8]. Although the bio-logger can focus more on target species or individuals, it cannot record enormous amounts of data like the ABR system on camera traps can. Recent bio-loggers have been equipped with solar panels (e.g., [19]). However, it would be difficult for solar panels to cover the large current consumption due to video recordings (more than 100 mA). Yet, the battery life can be elongated either by using a larger battery or adjusting logging parameters. We used a 500 mAh battery, considering the weight of the bio-logger relative to black-tailed gulls' body mass. For larger animals, larger batteries can be installed. Video recordings and GPS data acquisition consume much more battery than other sensors, such as accelerometers. Our bio-loggers are highly programmable, allowing users to configure settings such as enabling or disabling the camera and/or GPS, adjusting the camera recording time, or setting the GPS data sampling rate. It is possible to record only sound using the microphone when one chooses not to use a camera, which will reduce battery consumption and increase the number of playback sessions.

In addition, the multifunctionality of the system results in a slightly increased weight. The inclusion of a camera and speaker also adds to its height. Therefore, the shape of the logger should be optimised to reduce aerodynamic drag, and its overall size and weight should also be improved.

The field experiments also highlighted several hardware and/or software stability issues. First, there was a case where the bio-logger's program froze and lost control of the camera module, and it continued to record videos for more than 40 min (LBP01 S03). Reverse engineering revealed that resin for waterproofing had seeped into the camera's connector area, most likely causing an unstable connection. To prevent this issue, we updated our waterproofing methods, covering the camera's connector area with a liquid gasket before waterproofing with resin.

Second, the GPS module of the bio-logger could be unstable. During the development of the bio-logger, we tried to reconcile the two inherently conflicting goals of multifunctionality and miniaturisation. Therefore, the predecessor to the current version had noise sources around the GPS module, which significantly reduced the sensitivity of the GPS module. Although the current version has managed to mitigate the noise problem by carefully placing the parts on the board, especially around the GPS module. However, it still required careful operation. Generally, it takes some time (typically 1–2 minutes) for the GPS module to first fix the position after rebooting (cold start), and it can be difficult to fix the position if the GPS module is moving very fast at this stage. In some of the field trials, a sleep function was used to save battery consumption at nighttime; they were programmed to complete the GPS position fix before starting data logging after rebooting from the sleep mode. There was a case where the position fix was not completed for more than three hours. This is probably because the bird was on the move (e.g., flying back to the colony at high speed) when the bio-logger rebooted, and the battery was consumed a lot without fixing the position. Software measures were needed, such as a backup algorithm to deal with such situations (e.g., if the position

is not fixed for 5 minutes after rebooting, it goes back into sleep mode and waits for 30-60 minutes until the next reboot). In the future, adding an external antenna to the GPS module may be possible, which would increase the GPS sensitivity. We have already performed preliminary tests on this point and confirmed that the GPS sensitivity will be greatly improved using an external antenna. Increased GPS sensitivity would also allow for more flexibility in changing the sampling rate of GPS data to improve the battery life further. We plan to update hardware and software to overcome the above-mentioned and other potential limitations.

Text S12. Further results and discussion on the birds' responses to audio playbacks

In the main text, we intended to focus on the methodological aspects of the proposed system, due to the word limit. Therefore, we did not include the detailed results and discussion on the black-tailed gulls' responses to audio stimuli. In this supplementary section, we provide further results and discussion on the responses of the gulls.

Gulls' responses observed in this study

As described in the main text, our analysis demonstrated that several gulls showed clear responses to audio stimuli (at least in LBP01 S00; LBP01 S02; LBP03 S00; LBP03 S01; LBP06 S00; and LBP06 S01). Among the three response indicators used in this study, the results based on AD-MPR were easy to intuitively interpret because the spikes in the time-series sequences of AD-MPR were directly linked to the increase in the gulls' head (and neck) movements in the video data. The results based on AD-MPR are consistent with the first author's subjective observations from watching the video data. In addition, most of the changes in AD-MPR were positive ones (Figure 3f).

The previous studies have categorised the gulls' responses to audio stimuli into eight levels: levels range from 0 to 7, with higher numbers indicating a higher intensity of response [9, 13]. Level 0 indicates that gulls showed no response, and level 7 indicates that gulls showed escape behaviour from the nest including flight [9, 13]. Although it should be noted that the conditions (e.g., situations and duration of audio playback) of the playback experiments in these previous studies [9, 13] and the present study are different, the comparison may be useful to better understand the intensity of the gulls' response observed in the study. The playback effects measured using AD-MPR shrunk when using 10, 15, and 20 s windows, compared to those with 5 s windows (Figures S8 & S9), suggesting that the vigilance or scanning behaviours lasted during the playback and/or until a bit after the end of playback. The responses observed in this study might be categorised as those similar to response levels 1 or 2 in the previous studies on gulls: level 1 indicates that gulls first showed increased vigilance and then relaxed during the playback period, while level 2 indicates that gulls showed increased vigilance during the entire playback period [9, 13]. Level 3 indicates that gulls engaged in extreme vigilance with their neck fully outstretched, and/or rapid scanning of threats, but it also involves the use of vocalisation [9, 13]. Although several responses (e.g., LBP03 S00) may be classified as extreme vigilance or rapid scanning behaviours, there were no signs of calling in response to playback in this study; thus, the responses may not reach level 3 or a different categorisation may be needed for the playback targeting flying gulls. Moreover, the Bayesian analysis on AD-MPR implied that there are individual variations in terms of response intensity or response threshold, as shown in Figure S14. It also

implied that there might be a habituation effect (Figure 3i; 89% BCI did not include zero while 97% BCI did).

On the other hand, interpreting the results based on S-VeDBA and AD-Speed required more caution compared to AD-MPR. This is because behavioural changes that were likely unrelated to the audio playback were more easily detected, particularly in our data analysis using S-VeDBA and AD-Speed. The changes in S-VeDBA and AD-Speed included both positive and negative ones and the proportions of positive changes were almost similar to those of negative changes (Figure 3de). Here, we briefly describe some difficult cases. For instance, there was a case (LBP05 S00) where the bird performed a quick, intense movement just before the playback start timing. The movement involved high amplitude acceleration signals, increasing S-VeDBA values before the playback. An intense head-shaking was also observed after the audio playback start timing in another case (LBP07 S03), significantly influencing S-VeDBA values after the playback as well as AD-MPR values. In this case, the head-shaking occurred just after a short vigilance-like behaviour. Although the short vigilance-like behaviour might be a response to the playback, we are not sure if the head-shaking behaviour was also part of the response. In addition, there were cases (LBP03 S08; LBP08 S13) where the bird stopped flying after the video recording started but started flying again when the bio-logger was in the playback standby mode; the bio-logger started the playback soon (about 6 seconds) after the bird started flying. In this case, intense flapping for take-off resulted in acceleration signals with larger amplitudes, increasing S-VeDBA values before the playback. This also influenced the ground speed, thereby affecting AD-Speed, especially for LBP03 S08. For LBP08 S13, the bird's head was in the frames during intense flapping for take-off, affecting AD-MPR before the playback. Moreover, it is worth noting that even when the speed decreases, AD-Speed values will be large if the magnitude of change is significant. There was a case (LBP05 S07) where the ground speeds decreased before the playback for some reason, resulting in larger AD-Speed values. In the case of LBP06 S04, the ground speed decreased after the playback, and the AD-Speed values increased accordingly. We recognise that these cases may have added noise to our dataset. Hopefully, increasing the sample size or updating the algorithm to avoid playing soon after the birds change their speed or perform head-shaking would reduce the noise in future studies. More importantly, these cases highlight that collecting multimodal data is important.

Even when clear responses were observed in AD-MPR, there were not necessarily clear changes in S-VeDBA and AD-Speed (e.g., LBP06 S01), while there was an extreme case where we observed clear changes in all three indicators (LBP03 S00 as shown in Figure 4def in the main text). A previous study on gulls demonstrated that audio stimuli may alone elicit responses with higher intensity if the perceived threat levels imposed by audio stimuli are high enough [13]. Overall, it is likely that our audio stimuli have imposed the threat to the extent of eliciting vigilance and scanning behaviours for some birds (not for all the birds) but not to the extent of eliciting anti-predator or escape behaviours. In addition, a study on black-tailed gulls argued that the responses (to conspecific alarm calls) were intensified if the gulls visually detected a predator [10]. In this study, since gulls likely did not detect any threats by vigilance or scanning after perceiving audio stimuli, they might not have exhibited subsequent anti-predator or escape behaviours, which should be associated with clear changes in acceleration data and moving speed.

Why clear responses were observed only in limited numbers of sessions

Although we have focused on sessions where clear responses were observed in the above subsections, such playback sessions were limited in numbers. Here, we raise and discuss some of the potential factors that may have affected the results.

1. Environmental noise: Due to the weight constraints of bio-loggers for black-tailed gulls, we could only use a small speaker and the maximum volume that could be played by the speaker was limited. Although we believe that 70–90 dB (measured at a 15 cm distance, a distance from back to ears, see Text S4) would be sufficient to be perceived by the gulls under normal circumstances, loud environmental noise sounds may have reduced the relative volume of playback sound. For instance, when the wind speeds were very high, the wind noise around the birds may have diminished the relative volume of playback sound.

2. Individual variation: As indicated in Figure S14c, there may be individual variation, if not very clear, in thresholds for responding and/or in the intensity of the response. Assuming there are indeed individual differences, we remain uncertain about the factors shaping them and this would be an interesting point to explore in future studies. For instance, if data can be collected from many individuals, it might be possible to analyse the relationship between individual characteristics and response intensity.

3. Habituation effect: As shown in Figures 3i and S10, there was a tendency for the birds' responses (vigilance or scanning behaviours) to diminish or disappear entirely as the number of audio playback counts increased.

4. Length (duration) of audio data: The duration of audio files used in the previous playback studies on gulls [9, 13, 2] were longer (30, 60, and 30 s, respectively) than that used in the present study (4.2 seconds). The use of longer audio files may ensure that the audio stimuli are perceived by the birds even when the loud environment noise reduces the relative volume of playback sound, for instance.

5. Type of audio data: The previous studies have demonstrated that gulls showed clear responses to conspecific alarm calls [9, 13, 2]. In particular, one study demonstrated that conspecific alarm calls elicited response intensities that were similar to those induced by predator calls [9]. Given that predators would not normally produce calls while pursuing prey in flight, the use of conspecific alarm calls would elicit more biologically meaningful responses from the gulls. Since our bio-loggers can be programmed to play any type of audio data, it is feasible to test the idea in future experiments as long as good-quality audio data are available.

6. Quality of audio data: As discussed in the main text, the sounds played by a bio-logger on the bird's back might be unnatural. This is because gulls are more likely to respond to distant predator calls in the natural environment, not those perceived directly from such proximity. Since how the sound is perceived by the subject is critical in playback experiments, adjusting playback sound to mimic a distant predator call more accurately would be important in future experiments. For instance, by

gradually adjusting the volume, it may be possible to mimic how a gull perceives the distant call of a predator in flight.

In conclusion, we recognise that there is plenty of room for improvement of the proposed, autonomous playback system using bio-loggers; we plan to refine the system and/or audio data and test them in future experiments. It would be relatively easy to test different lengths and types of audio data. Improving the quality of audio data to mimic realistic sounds would be a bit challenging but could be implemented. A more challenging but interesting future direction would be the development of a feedback system, which allows the bio-loggers to flexibly or interactively [e.g., 7] change audio data types and their parameters as well as the target situation of playback depending on the responses of a subject animal to audio stimuli. Such a flexible autonomous audio playback experiment system in the wild is unprecedented, and more exploratory research is needed, but if refined, it could be an innovative tool that expands the possibilities of animal behaviour and ecology research as well as other relevant fields.

Supplementary Data

Supplementary Figures

Figure S1

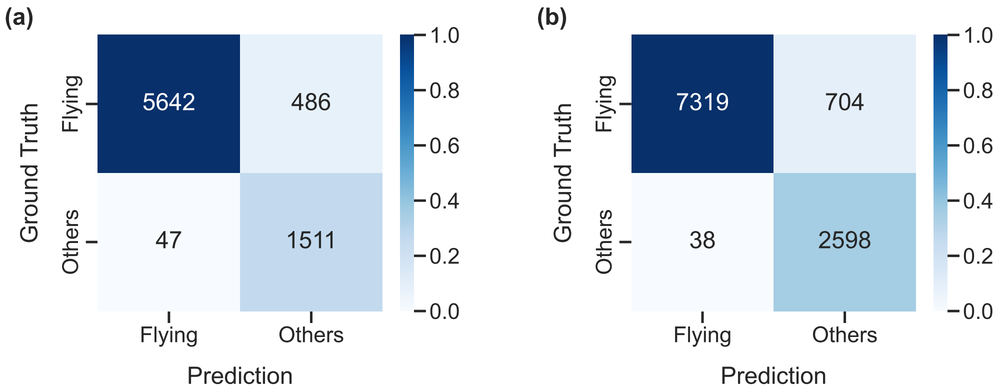

Figure S1. Confusion matrix showing the binary classification performance (flying or others i.e. not-flying behaviours) of machine learning (Decision Tree) models. Results from (a) test data and (b) field experiment data.

Figure S2

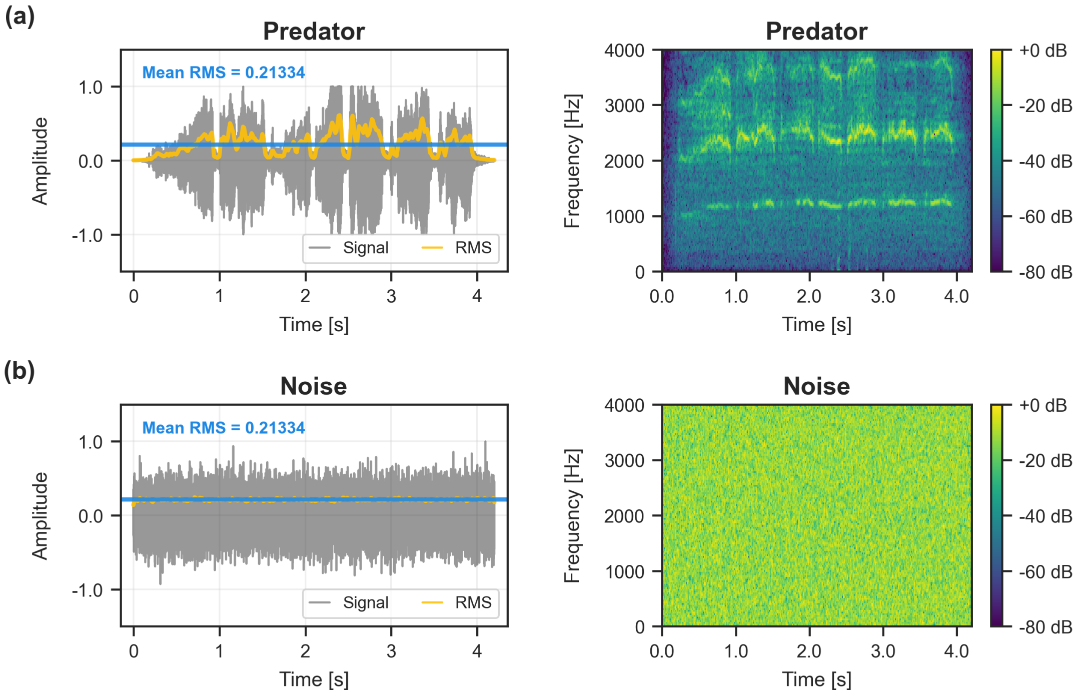

Figure S2. A waveform and spectrogram of audio data used in the field experiments: (a) predator call and (b) white noise.

Figure S3

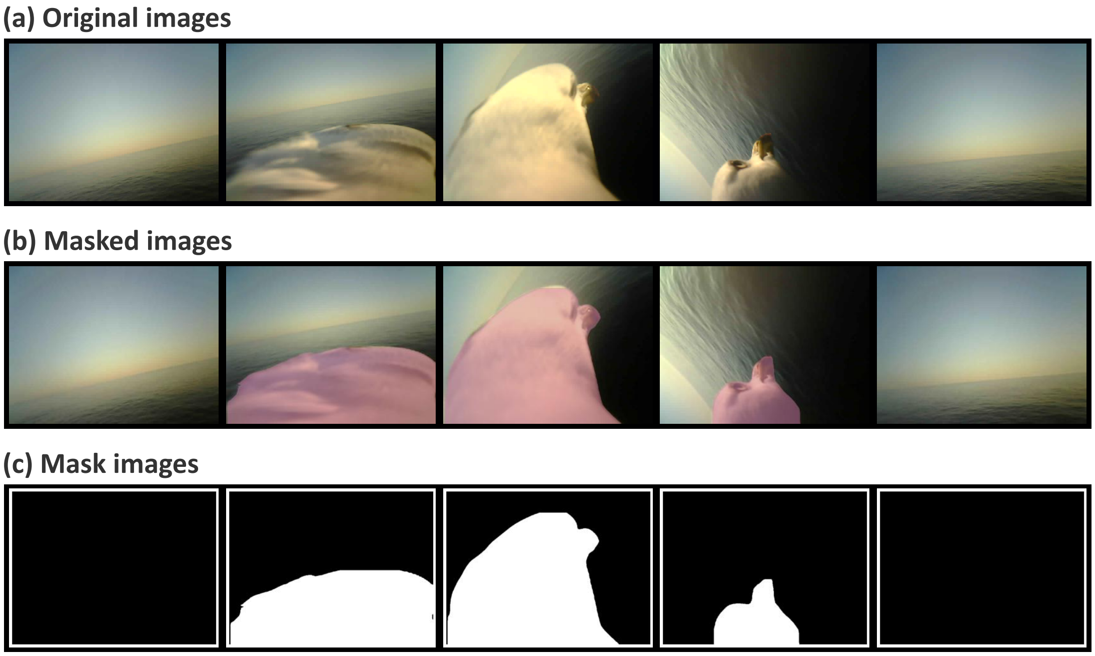

Figure S3. Visualisation of image segmentation using custom-trained YOLOv8 model: (a) original images; (b) masked images; and (c) mask images in black and white.

Figure S4

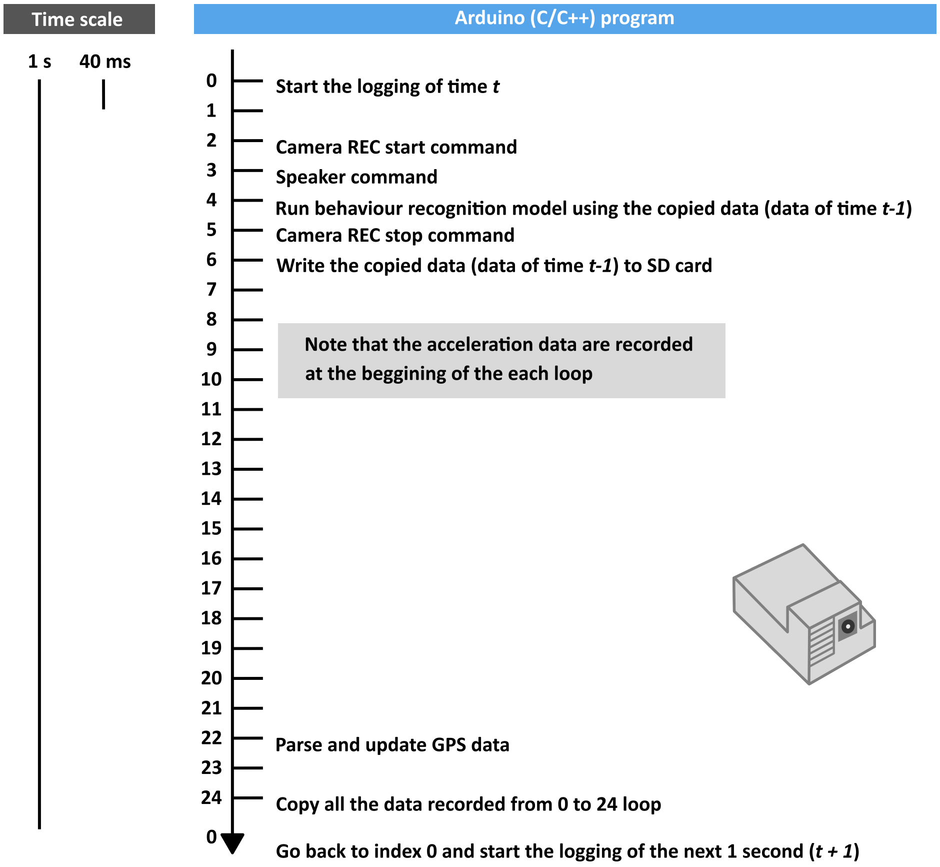

Figure S4. Schematic overview of the data logging routine in the Arduino program for our bio-logger, showing the timing of issuing camera/speaker-related commands, updating acceleration and GPS data, and writing the logging data to the SD card.

587 ## Figure S5

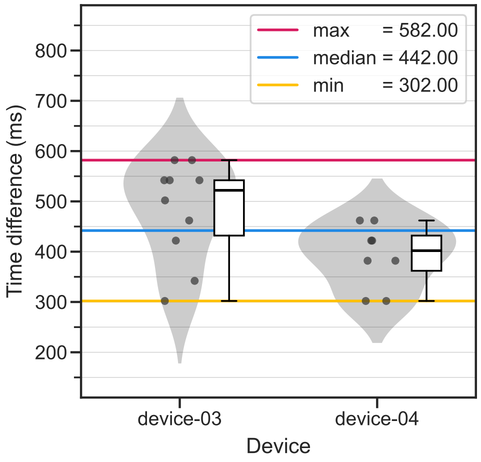

Figure S5. Distribution of estimated time differences between the timing of issuing the audio playback start command and the timing of actual sound being produced by the speaker.

588

Figure S6

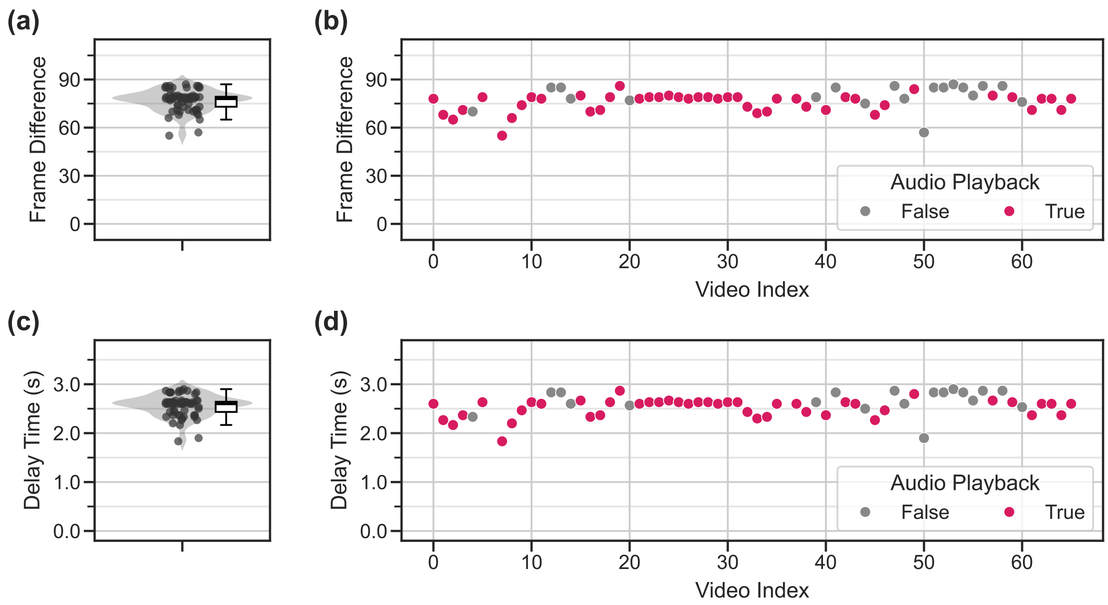

Figure S6. Differences in the expected and actual numbers of frames (a, b) and the corresponding times in seconds (c, d) for each video data.

Figure S7

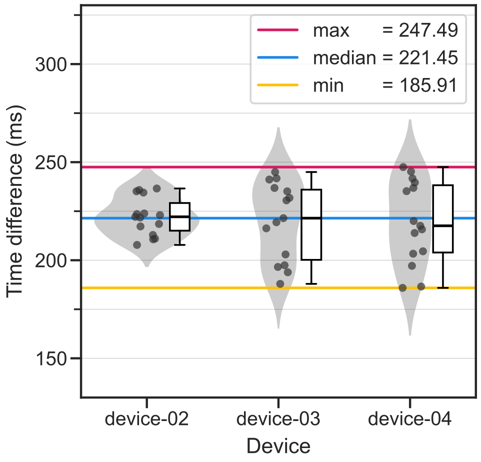

Figure S7. Distribution of estimated time differences between the timing of handclaps and the timing of their sounds in video data.

590 **Figure S8**

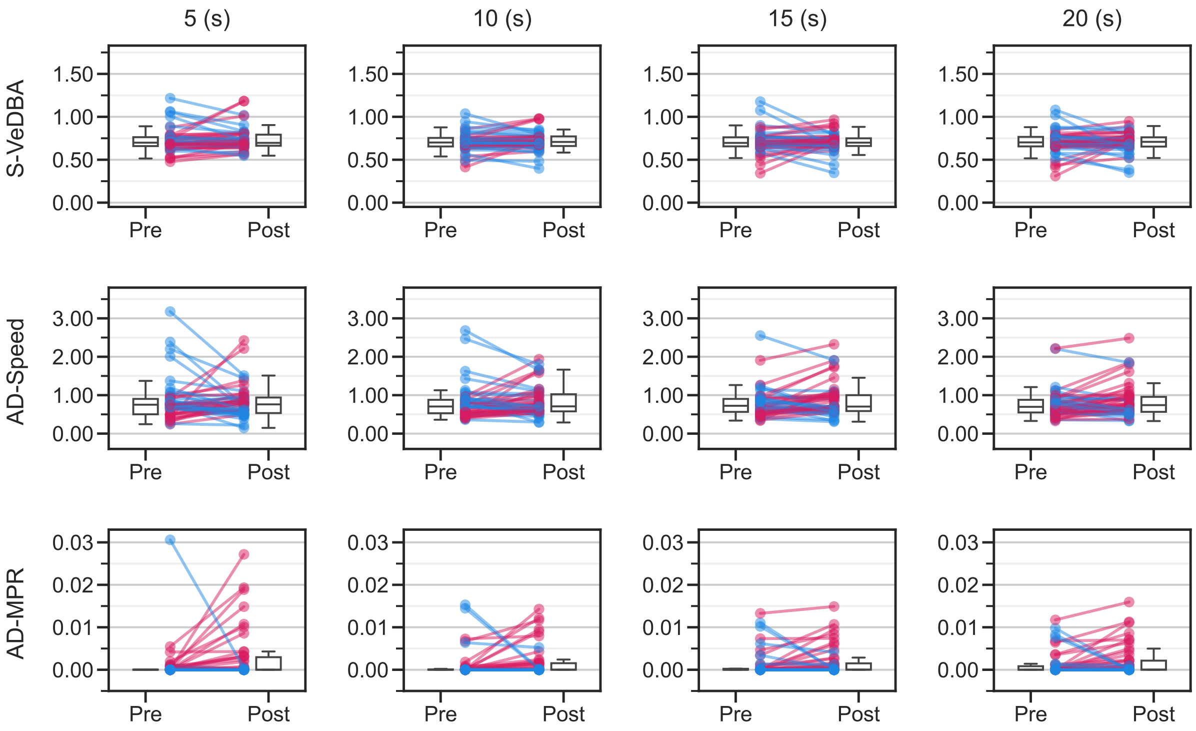

Figure S8. Comparison of mean response indicator values during pre- and post-periods of audio playback (5, 10, 15, and 20 seconds). The vertical axis shows the mean values of one of the three response indicators, and each slope line connects the two data points (pre-mean and post-mean) from each audio playback session. The first, second, and third rows show the results for each of the three response indicators: the vectorial dynamic body acceleration (VeDBA) smoothed over 2 seconds (S-VeDBA; based on 25 Hz tri-axial acceleration data); the absolute difference in ground speeds between successive seconds (AD-Speed; based on 1 Hz GPS data); and the absolute difference in masked pixel ratios (MPRs) between two successive frames (AD-MPR; based on 30 FPS video data), respectively.

591 # Figure S9

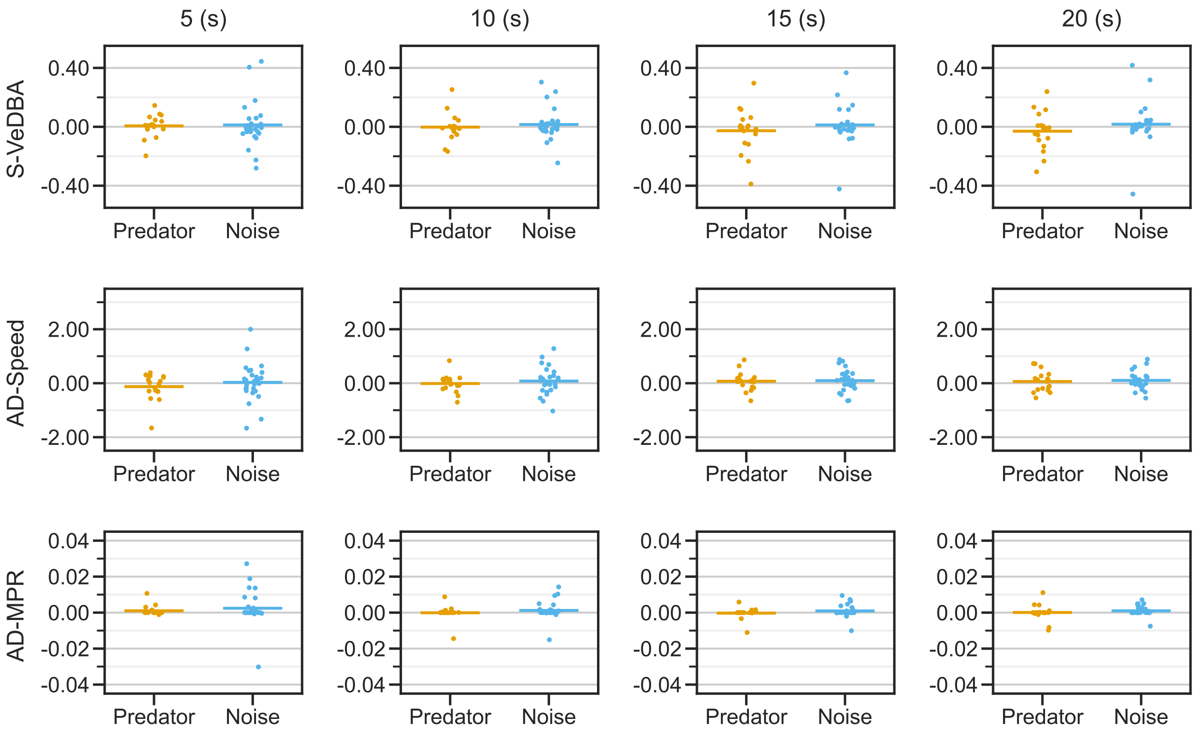

Figure S9. Comparison of differences in mean response indicator values during pre- and post-periods of audio playback (5, 10, 15, and 20 seconds) per audio data type (predator call or white noise). The response indicators are S-VeDBA, AD-Speed, and AD-MPR as described in Text S5. The pre-mean value was subtracted from the post-mean value for each audio playback session. The vertical axis shows the difference in the average values of one of the three response indicators between 5-, 10-, 15-, and 20-second-long pre- and post-periods. A horizontal line (orange or light blue) shows the average of difference values for each category in each plot.

592 **Figure S10**

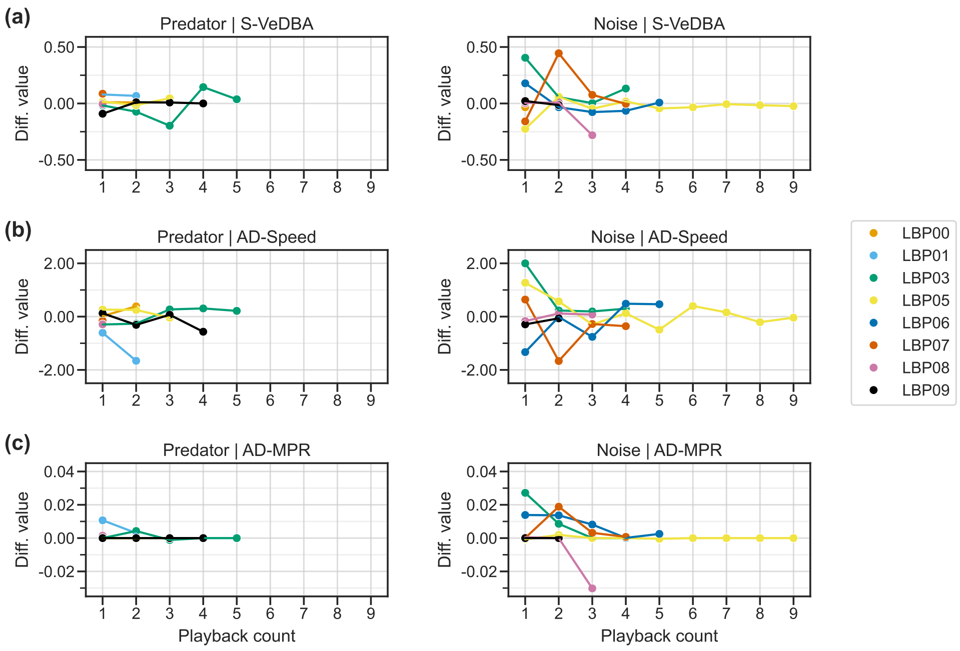

Figure S10. Relationship between the number of playback counts and the differences in mean response indicator values (the vertical axis) during the pre- and post-periods of audio playback (5 seconds) per audio data type (predator call or white noise). The response indicators are S-VeDBA (a), AD-Speed (b), and AD-MPR (c) as described in Text S5. The horizontal axis of each plot indicates the number of playbacks per bird and audio data type.

593 # Figure S11

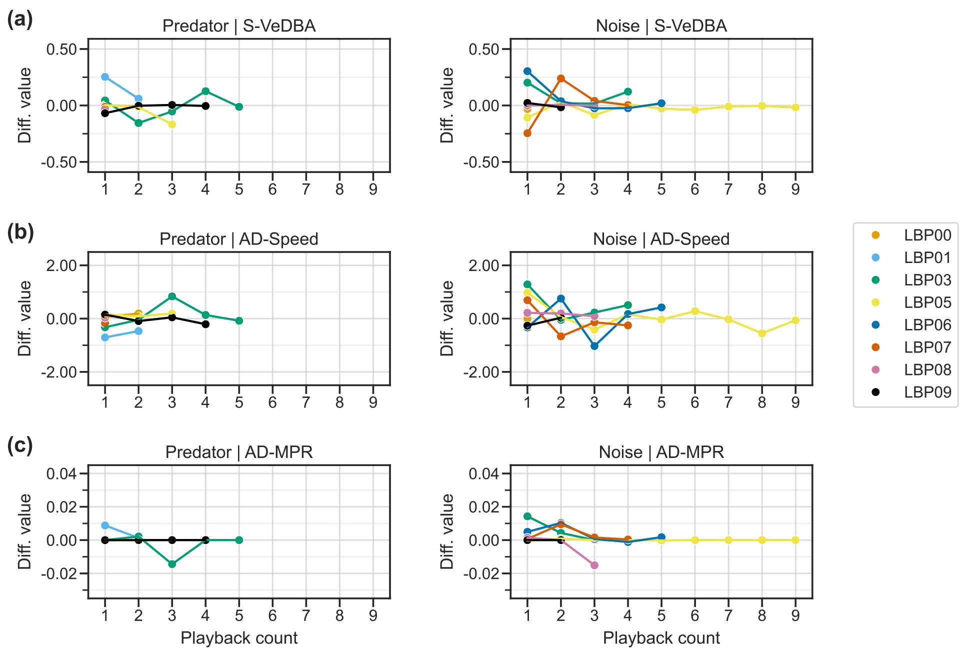

Figure S11. Relationship between the number of playback counts and the differences in mean response indicator values (the vertical axis) during the pre- and post-periods of audio playback (10 seconds) per audio data type (predator call or white noise). The response indicators are S-VeDBA (a), AD-Speed (b), and AD-MPR (c) as described in Text S5. The horizontal axis of each plot indicates the number of playbacks per bird and audio data type.

594 **Figure S12**

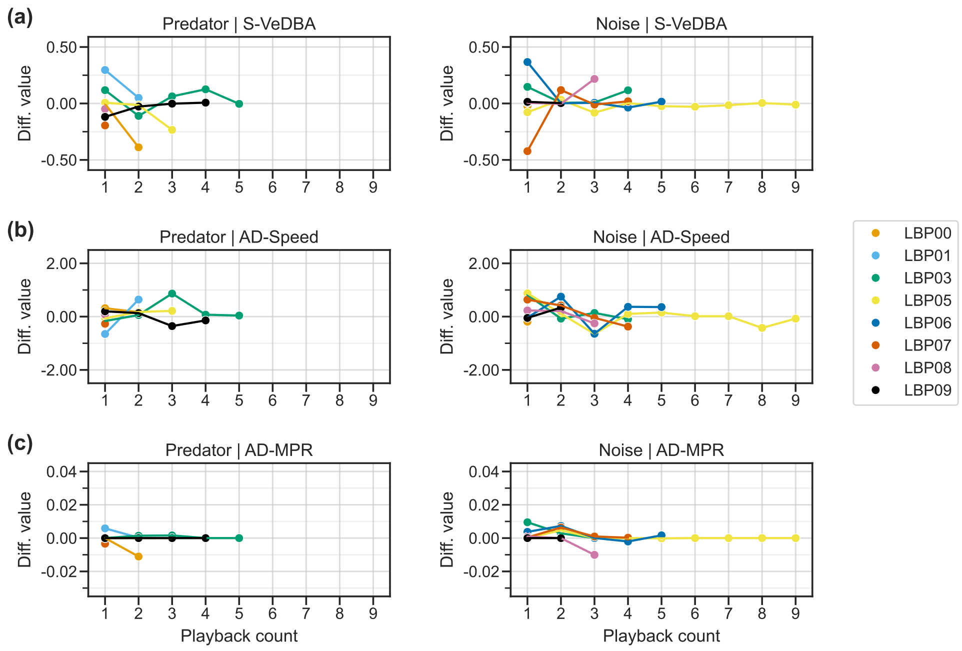

Figure S12. Relationship between the number of playback counts and the differences in mean response indicator values (the vertical axis) during the pre- and post-periods of audio playback (15 seconds) per audio data type (predator call or white noise). The response indicators are S-VeDBA (a), AD-Speed (b), and AD-MPR (c) as described in Text S5. The horizontal axis of each plot indicates the number of playbacks per bird and audio data type.

595 # Figure S13

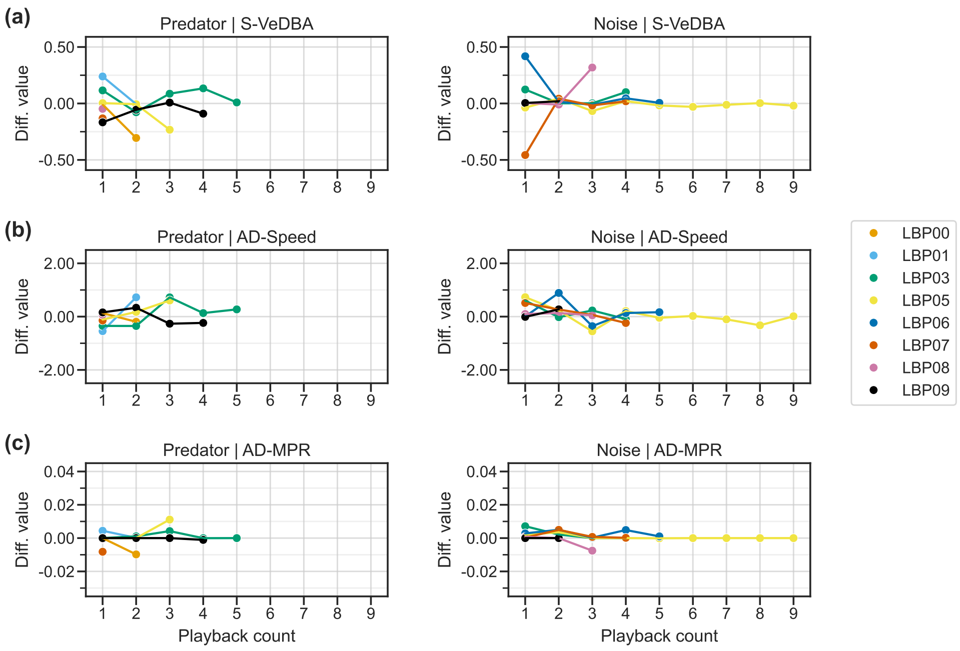

Figure S13. Relationship between the number of playback counts and the differences in mean response indicator values (the vertical axis) during the pre- and post-periods of audio playback (20 seconds) per audio data type (predator call or white noise). The response indicators are S-VeDBA (a), AD-Speed (b), and AD-MPR (c) as described in Text S5. The horizontal axis of each plot indicates the number of playbacks per bird and audio data type.

596 # Figure S14

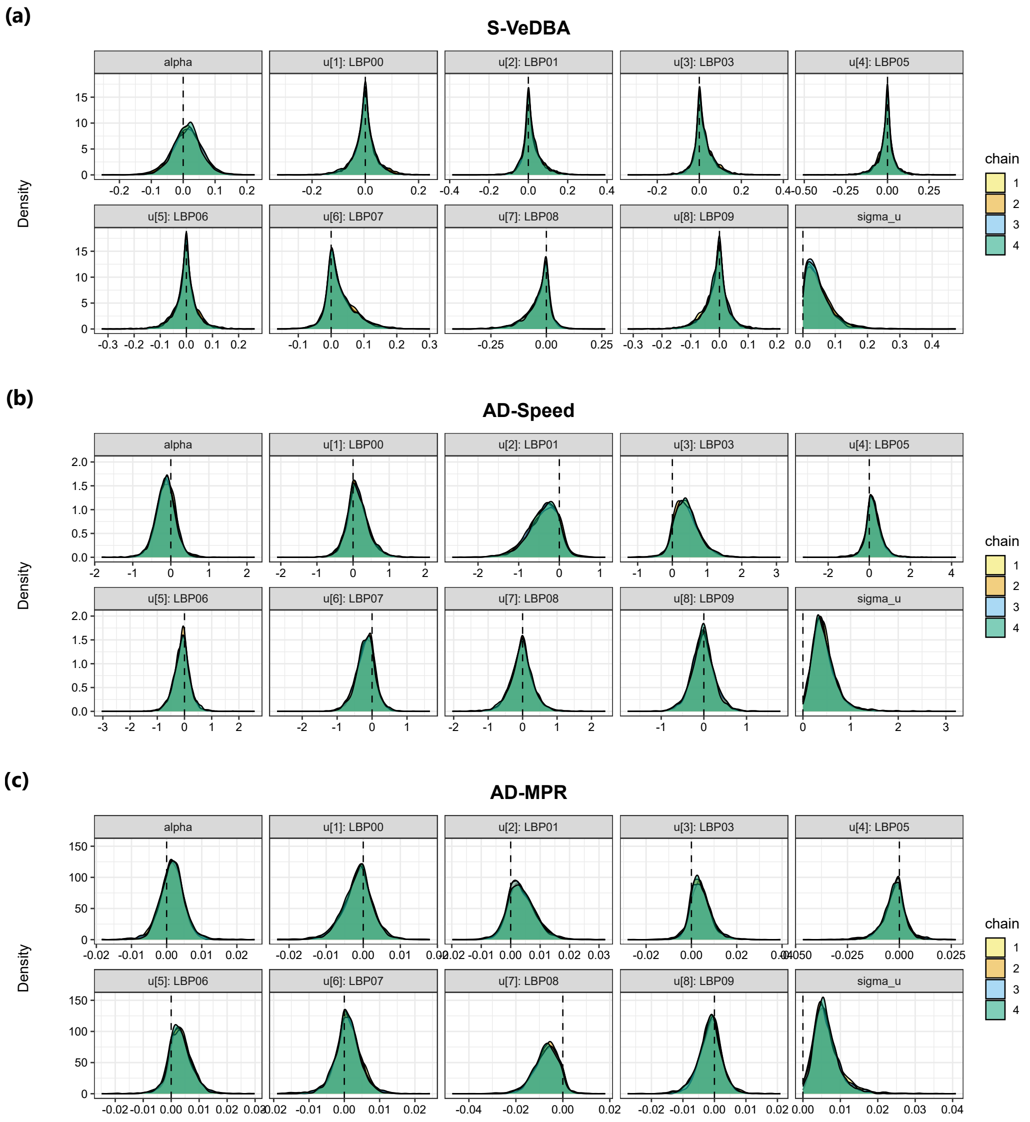

Figure S14. Posterior distributions of the global intercept parameter (alpha), the individual intercept parameters ($u[1]$–$u[8]$), and the standard deviation parameter (σ_u) of $u_{[k]}$ from models (Text S9) for the three response indicators, S-VeDBA (a), AD-Speed (b), and AD-MPR (c).

597 # Figure S15

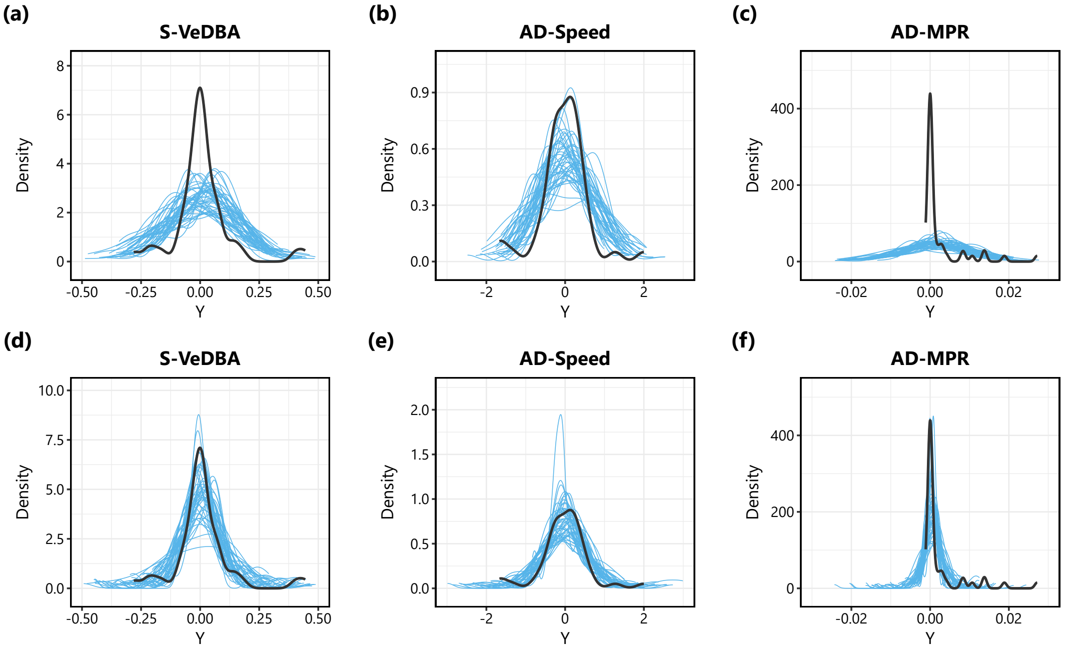

Figure S15. Posterior predictive checks of models that used normal distribution (a, b, c) and models that used Student-t distribution with the degree of freedom of 2.2 (d, e, f). Light blue lines in each plot represent 100 posterior predictive distributions that were randomly drawn using each model, while the black line represents the probability distribution of the observed data.

References

[1] Brodersen, K.H., Gallusser, F., Koehler, J., Remy, N. & Scott, S.L. (2015) Inferring causal impact using bayesian structural time-series models. *Annals of Applied Statistics*, **9**, 247–274. https://dx.doi.org/10.1214/14-AOAS788.

[2] Di Giovanni, J., Fawcett, T.W., Templeton, C.N., Raghav, S. & Boogert, N.J. (2022) Urban gulls show similar thermographic and behavioral responses to human shouting and conspecific alarm calls. *Front Ecol Evol*, **10**. https://dx.doi.org/10.3389/fevo.2022.89198510.3389/fevo.2022.891985.s001.

[3] Dwyer, B., Nelson, J. & Hansen, T. (2024) Roboflow (version 1.0).

[4] Flower, T.P., Gribble, M. & Ridley, A.R. (2014) Deception by flexible alarm mimicry in an african bird. *Science*, **344**, 513–516. https://dx.doi.org/10.1126/science.1249723.

[5] Jain, A., Bansal, R., Kumar, A. & Singh, K.D. (2015) A comparative study of visual and auditory reaction times on the basis of gender and physical activity levels of medical first year students. *Int J Appl Basic Med Res*, **5**, 124–127. https://dx.doi.org/10.4103/2229-516X.157168.

[6] Jocher, G., Chaurasia, A. & Qiu, J. (2023) Ultralytics YOLO (version 8.0.0).

[7] King, S.L. (2015) You talkin' to me? interactive playback is a powerful yet underused tool in animal communication research. *Biol Lett*, **11**, 20150403. https://dx.doi.org/10.1098/rsbl.2015.0403.

[8] Korpela, J., Suzuki, H., Matsumoto, S., Mizutani, Y., Samejima, M., Maekawa, T., Nakai, J. & Yoda, K. (2020) Machine learning enables improved runtime and precision for bio-loggers on seabirds. *Commun Biol*, **3**, 633. https://dx.doi.org/10.1038/s42003-020-01356-8.

[9] MacLean, S.A. & Bonter, D.N. (2013) The sound of danger: threat sensitivity to predator vocalizations, alarm calls, and novelty in gulls. *PLoS One*, **8**, e82384. https://dx.doi.org/10.1371/journal.pone.0082384.

[10] Park, S.R., Chung, H., Cheong, S.W., Lee, S.Y. & Sung, H.C. (2007) Anti-predator responses of black-tailed gull (larus crassirostris) flocks to alarm calls during the post-breeding season. *J Ecol Field Biol*, **30**, 9–15.

[11] Qasem, L., Cardew, A., Wilson, A., Griffiths, I., Halsey, L.G., Shepard, E.L.C., Gleiss, A.C. & Wilson, R. (2012) Tri-axial dynamic acceleration as a proxy for animal energy expenditure; should we be summing values or calculating the vector? *PLoS One*, **7**, e31187. https://dx.doi.org/10.1371/journal.pone.0031187.

[12] R Core Team (2022) R: A language and environment for statistical computing.

[13] Shah, S.S., Greig, E.I., MacLean, S.A. & Bonter, D.N. (2015) Risk-based alarm calling in a nonpasserine bird. *Anim Behav*, **106**, 129–136. https://dx.doi.org/10.1016/j.anbehav.2015.05.011.

[14] Shepard, E.L.C., Wilson, R.P., Halsey, L.G., Quintana, F., Gómez Laich, A., Gleiss, A.C., Liebsch, N., Myers, A.E. & Norman, B. (2008) Derivation of body motion via appropriate smoothing of acceleration data. *Aquat Biol*, **4**, 235–241. https://dx.doi.org/10.3354/ab00104.

[15] Stan Development Team (2019) Stan modeling language users guide and reference manual.

[16] Stan Development Team (2021) RStan: the R interface to stan.

[17] Tomita, N., Mizutani, Y., Fujii, H., Sugiura, R., Yanai, T., Asano, M. & Niizuma, Y. (2010) Mortality of adult black-tailed gulls *Larus crassirostris* on kabu island, aomori prefecture. *Jpn J Ornithol*, **59**, 80–83. https://dx.doi.org/10.3838/jjo.59.80.

[18] Wilson, R.P., Börger, L., Holton, M.D., Scantlebury, D.M., Gómez-Laich, A., Quintana, F., Rosell, F., Graf, P.M., Williams, H., Gunner, R., Hopkins, L., Marks, N., Geraldi, N.R., Duarte, C.M., Scott, R., Strano, M.S., Robotka, H., Eizaguirre, C., Fahlman, A. & Shepard, E.L.C. (2020) Estimates for energy expenditure in free-living animals using acceleration proxies: A reappraisal. *J Anim Ecol*, **89**, 161–172. https://dx.doi.org/10.1111/1365-2656.13040.

[19] Yu, H., Deng, J., Leen, T., Li, G. & Klaassen, M. (2022) Continuous on-board behaviour classification using accelerometry: A case study with a new GPS-3G-bluetooth system in pacific black ducks. *Methods Ecol Evol*, **13**, 1429–1435. https://dx.doi.org/10.1111/2041-210x.13878.
